# PathoScope 2.0: a complete computational framework for strain identification in environmental or clinical sequencing samples

**DOI:** 10.1186/2049-2618-2-33

**Published:** 2014-09-05

**Authors:** Changjin Hong, Solaiappan Manimaran, Ying Shen, Joseph F Perez-Rogers, Allyson L Byrd, Eduardo Castro-Nallar, Keith A Crandall, William Evan Johnson

**Affiliations:** 1 Computational Biomedicine, Boston University School of Medicine, 72 E Concord St. E645, Boston, MA 02118, USA; 2 Bioinformatics Program, Boston University, Boston, MA 02125, USA; 3 Computational Biology Institute, George Washington University, Ashburn, VA 20147, USA

## Abstract

**Background:**

Recent innovations in sequencing technologies have provided researchers with the ability to rapidly characterize the microbial content of an environmental or clinical sample with unprecedented resolution. These approaches are producing a wealth of information that is providing novel insights into the microbial ecology of the environment and human health. However, these sequencing-based approaches produce large and complex datasets that require efficient and sensitive computational analysis workflows. Many recent tools for analyzing metagenomic-sequencing data have emerged, however, these approaches often suffer from issues of specificity, efficiency, and typically do not include a complete metagenomic analysis framework.

**Results:**

We present PathoScope 2.0, a complete bioinformatics framework for rapidly and accurately quantifying the proportions of reads from individual microbial strains present in metagenomic sequencing data from environmental or clinical samples. The pipeline performs all necessary computational analysis steps; including reference genome library extraction and indexing, read quality control and alignment, strain identification, and summarization and annotation of results. We rigorously evaluated PathoScope 2.0 using simulated data and data from the 2011 outbreak of Shiga-toxigenic *Escherichia coli* O104:H4.

**Conclusions:**

The results show that PathoScope 2.0 is a complete, highly sensitive, and efficient approach for metagenomic analysis that outperforms alternative approaches in scope, speed, and accuracy. The PathoScope 2.0 pipeline software is freely available for download at: http://sourceforge.net/projects/pathoscope/.

## Background

The rapid and accurate characterization of microbial flora in clinical or environmental samples is critical for many applications including understanding the role of the microbiome in human health, personalized responses to acute or chronic infections, and early detection in disease outbreaks. With the steadily increasing number of microbial genomes available in public data repositories, metagenomic characterization using high-throughput sequencing techniques can be used to catalogue microbes co-habituating in human systems [1] and to rapidly identify pathogens responsible for infectious disease outbreaks [2-4]. This proliferation of metagenomic sequence data has resulted in the development of novel analytical approaches. Feature selection approaches exploit features from genomic patterns or composition [5,6], preserved sequence segments [7-9] or predetermined clade markers [10,11]. Assembly-based methods [12-15] have recently gained in popularity due to their increased sensitivity for strain identification. However, these approaches can suffer from issues of specificity, efficiency, and typically do not include a complete metagenomic analysis framework with reference library generation, read quality control, and reporting.

Here we present a complete framework for rapid and accurate metagenomic profiling at the subspecies level that overcomes the limitations of other approaches. In our previous work, we developed a statistical algorithm (formerly PathoScope 1.0 and henceforth denoted as the PathoID module of the PathoScope 2.0 framework) to reassign ambiguously aligned sequencing reads and accurately estimate read proportions from each genome in the sample [16]. In PathoScope 2.0, we have introduced performance improvements to the PathoID algorithm such as better utilization of alignment scores, the inclusion of reference length and alignment quality into the reassignment model, and the addition of flexible priors for the read proportions and ambiguity penalties. In addition, PathoScope 2.0 extends the PathoID module into a complete workflow for analyzing data from clinical or environmental sequencing samples, with novel modules that: (1) automatically extract custom reference genome libraries for microbial or host genomes (PathoLib); (2) construct reference indices, align reads, and filter reads that align to the host (PathoMap); (3) conduct complete, parallel read quality processing (PathoQC); (4) annotate all sequences in the reference library with information such as organism name, taxonomic lineage, and gene loci (PathoDB); and (5) provide detailed reports on organisms, read coverage, genes, and gene products identified in the study (PathoReport). The modular PathoScope 2.0 framework minimizes interaction for users with weaker computational backgrounds, while providing experienced users the flexibility to conduct analysis steps outside of the pipeline and to develop new plug-in modules.

## Results and discussion

### New features in Pathoscope 2.0

**Figure 1:**
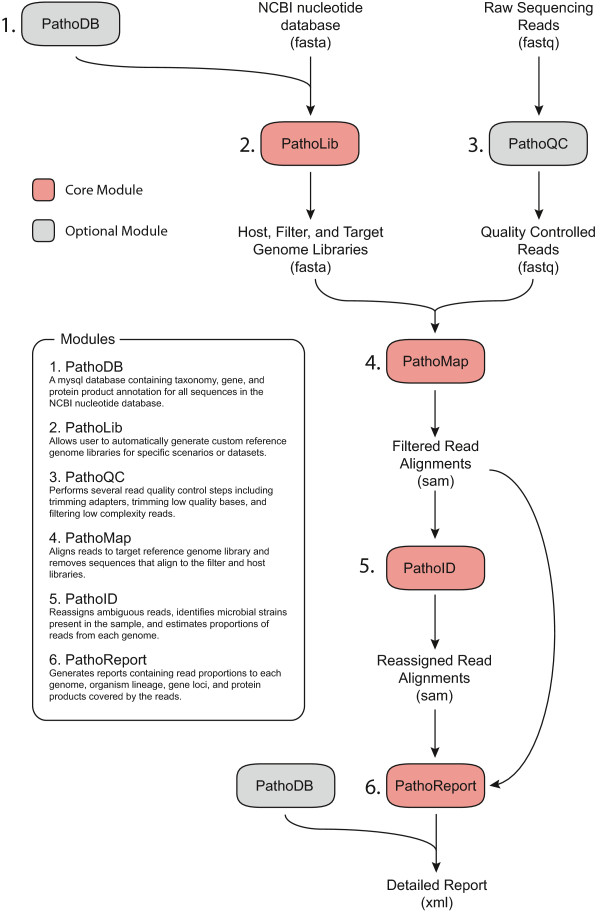
**Workflow of the PathoScope 2.0 framework.** PathoScope 2.0 consists of four core and two optional analysis modules for metagenomic profiling. Core modules: PathoLib extracts custom genome reference libraries, PathoMap aligns the reads to the reference library and filters host reads, PathoID identifies and estimates the proportions from each genome, and PathoReport provides detailed summary reports of the results. PathoDB provides additional annotation and PathoQC can be used to preprocess the reads prior to alignment.

With the introduction of PathoScope 2.0, we improve our previous PathoID algorithm for strain identification [16] and we extend our software into a complete framework for the analysis of metagenomic sequencing data. PathoScope 2.0 (Figure 1) currently consists of four core pipeline modules (PathoLib, PathoMap, PathoID, and PathoReport) and two optional ‘plug-in’ modules (PathoDB and PathoQC), with capability for seamless interaction with other metagenomic tools and for the development of future plug-in modules to increase the usability of the pipeline. Below are detailed descriptions of the novel features that are introduced in PathoScope 2.0:

### PathoLib: Automatic reference library extraction

**Figure 2:**
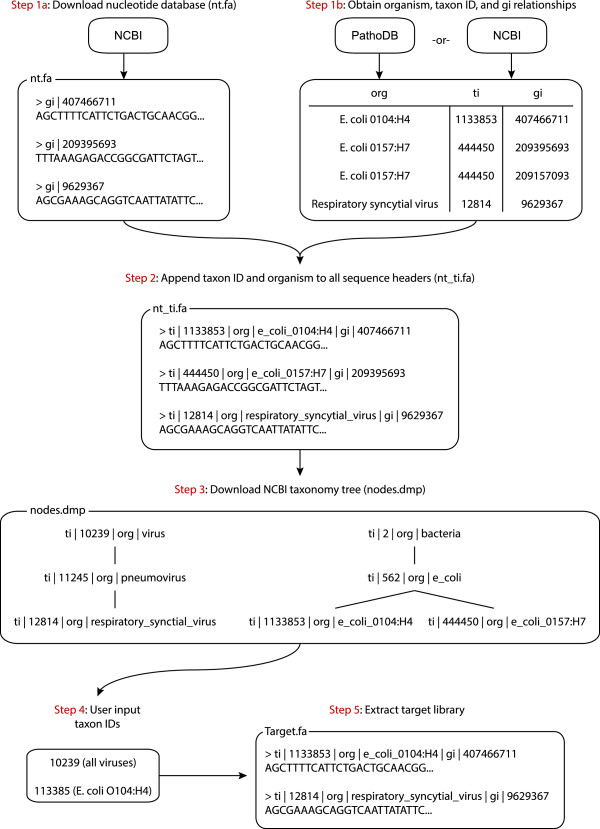
**PathoLib module workflow.** The PathoLib module will extract a reference library containing all genomes, chromosomes, transcripts, and other sequence fragments in the NCBI redundant nucleotide database associated user-defined taxonomic clade (NCBI taxID). If a higher-level taxID is given, PathoLib will optionally extract all sequences from lower-lever taxonomic designations based on the NCBI taxonomy tree.

The careful selection of a refined reference sequence library is crucial for all downstream analyses. The PathoLib module allows the user to automatically generate custom reference genome libraries for specific scenarios or datasets. The user supplies a set of NCBI taxonomy identification (taxID) numbers for organisms to be included in the library (Figure 2). The user can construct both a ‘target library’ (that is, pathogen genomes of interest) and a ‘filter library’ (for example, host genome or benign flora) for later use in the PathoMap module. The PathoLib module will extract all sequences in the NCBI nucleotide database associated with the taxIDs (for example, complete genomes, transcripts, plasmids, partially assembled fragments, and so on). In addition, if a high-level taxID is given (for example, kingdom, family, genus), PathoLib can also optionally extract all lower level sequences in the NCBI taxonomy tree. As PathoLib extracts the reference library, the NCBI GeneInfo number is linked to the taxID, and the taxID and organism name are appended to the sequence headers to further link sequences in downstream analyses.

### PathoMap: Efficient read alignment and filtering

**Figure 3:**
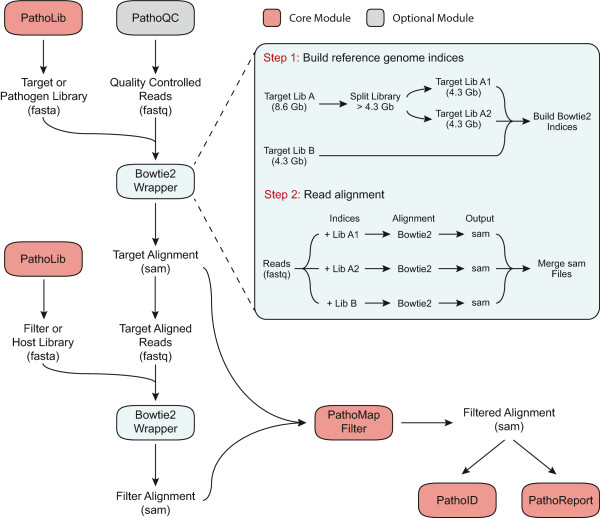
**PathoMap module workflow.** The PathoMap module aligns reads to the target library and removes any sequences that align to the filter library. PathoMap will: (1) index the reference library; (2) align the reads to the target library; and (3) filter any of the target-matching reads that also match the filter library. The current version of PathoMap includes a Bowtie 2 wrapper and allows users can conduct any part of the alignments outside the PathoMap framework.

The PathoMap module aligns reads to the target library and removes any sequences that align with an equal or greater score to the filter library (Figure 3). Inputs for this module are the raw read file (FASTQ) and both the target and filter reference libraries (FASTA format). PathoMap will: (1) index the reference library (splitting the library into multiple indices if necessary); (2) align the reads to the target library; and (3) filter any of the target-matching reads that also match the filter library. The current version of PathoMap includes a Bowtie 2 [17] wrapper (see Figure 3) with predetermined optimal alignment parameters for different read generation technologies (for example, Illumina: ‘--very-sensitive -k 100 --score-min L,-0.6,-0.6’; PacBio: ‘--very-sensitive -k 100 --score-min L, -0.6, -1.5’). The module also allows flexibility for the user to manually input Bowtie 2 parameters, or to conduct any part of the alignments outside the PathoMap framework by supplying an alignment file in SAM format (Li et al., [18]). Finally, the module is constructed in a way that wrappers for additional alignment algorithms can easily be substituted for the Bowtie 2 wrapper to accommodate diverse user preferences.

### PathoID: Reassignment of ambiguous reads

**Figure 4:**
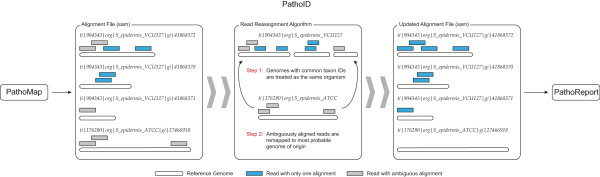
**PathoID module workflow.** The PathoID utilizes a penalized statistical mixture model to reassign ambiguous reads to the most likely source genome. The PathoID module also includes performance improvements to the originally published reassignment approach (PathoScope 1.0), by allowing users to calibrate prior information, better utilize alignment scores, and by including reference length and read alignment quality into the reassignment.

The PathoID module (Figure 4) comprises our previous PathoScope 1.0 software, which utilizes a penalized statistical mixture model that reassigns all ambiguous reads to the most likely source genome in the library [16]. PathoID takes as input either a SAM or BLAST (format 8) alignment file, and reassigns the reads to the most likely genome of origin. The PathoID module also includes three important performance improvements to the original PathoScope approach: (1) improving the utilization of alignment scores; (2) including both reference length and read alignment quality into the reassignment model; and (3) the addition of user-defined priors for read proportions and ambiguity penalties (See Materials and Methods for details). These improvements increase the number of reads that are correctly assigned to the source genome, reduce the number of false positive genomes identified, and allow PathoID to better handle cases where the reference is not fully assembled or the sample contains multiple substrains of the same species. PathoID produces an updated alignment file of read reassignments and a summary report containing genome-level read proportions.

### PathoReport: Detailed result reporting and annotation

**Figure 5:**
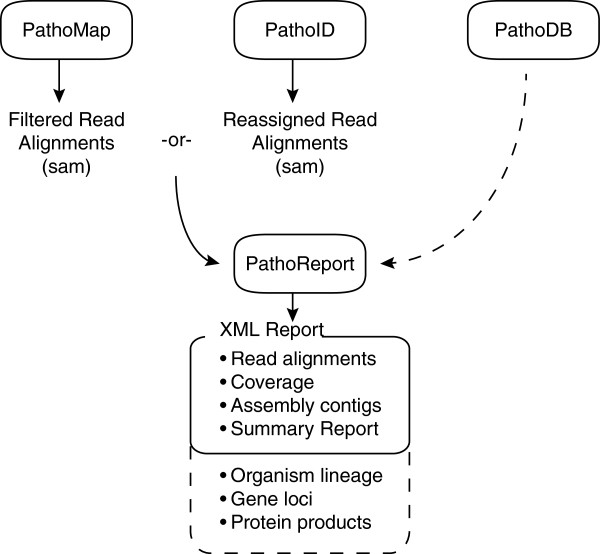
**PathoReport module workflow.** The PathoReport module outputs two report files including: (1) a tab-delimited (.tsv) report that contains a ranked list of genomes (with proportions) identified by the pipeline; and (2) an XML file containing detailed results including the reads assigned to each genome, contigs constructed from overlapping reads, and so on.

The PathoReport module (Figure 5) outputs two files from the pipeline. The first output file is a tab-delimited (.tsv) report that contains the genomes that were identified by the previous steps sorted by rank, along with high/low confidence read numbers and proportions assigned to each genome. The second file, in XML format, contains more detailed results, including the reads assigned to each genome and contiguous sequences (contigs) constructed from overlapping reads. In addition, in concert with the plug-in module PathoDB (described below), PathoReport will add additional annotation into the report such as organism lineage, gene loci, and protein products for genes covered by the reads. This XML output provides useful information for evaluating the quality of the results and facilitating downstream interpretation and analysis. For example, the specific reads assigned to a genome can be an important quality check for a metagenomic analysis to check if the reads are low complexity or contain multiple PCR duplicates. The contigs show the breadth of genomic coverage, can identify sequence variation from the reference, and facilitate scaffold-based genome assembly. The gene annotations identify the specific genes covered by the reads, can be use to annotation SNPs in specific genes, and (in RNA-seq studies) can identify which pathogenic genes are actively expressed. Examples of PathoReport XML files are given in Additional file 1.

### Optional plug-in modules

**Figure 6:**
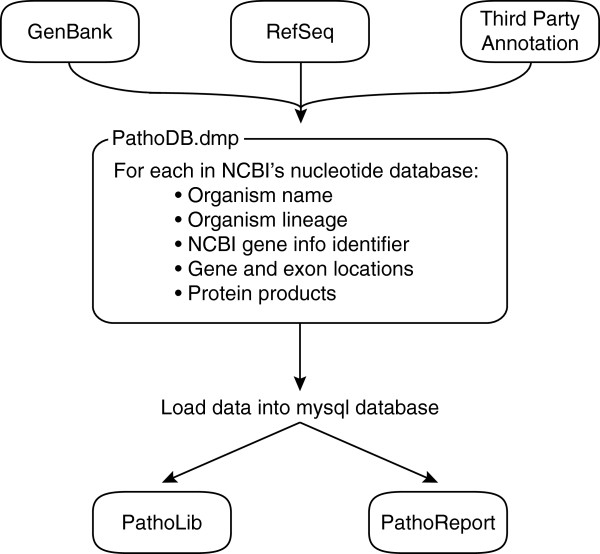
**PathoDB module workflow.** PathoDB is an optional module of pre-compiled annotation for all sequences in the NCBI nucleotide database. The PathoDB module automatically interacts with PathoReport to provide additional annotation in the detailed (XML) report such as organism lineage, gene loci, and protein products for any genes covered by the reads.

The PathoScope 2.0 framework was constructed to easily add modules to the pipeline. Currently, there are two available add-in modules: PathoQC and PathoDB. PathoQC is a read quality control module that performs several steps including trimming adapters, trimming low quality bases, and filtering low complexity sequences. PathoDB is a module consisting of taxonomy, gene, and protein product annotation for all sequences in the NCBI nucleotide database (Figure 6). In PathoDB, we have pre-compiled an annotation database containing information including organism name, lineage, NCBI GenInfo identifier, gene and exon locations, and protein products for all sequences in the NCBI nucleotide database. This information was assembled from 560 GB of annotation from the NCBI GenBank, RefSeq, and Third-Party Annotation resources (ftp://ftp.ncbi.nih.gov/). The PathoDB module automatically interacts with PathoReport to provide additional annotation in the detailed XML report. The compiled database is available for download from the PathoScope distribution webpage (http://sourceforge.net/projects/pathoscope/). In order to utilize PathoDB and automatically extract the database information in PathoReport, the user needs a MySQL client account or needs to set up a MySQL server. Because this requires several independent steps for installation, we have made this module optional.

Future plug-in modules will be added, including additional aligner wrappers, modules for data visualization, and modules for ‘post-diagnostic’ variant calling, annotation, and genome assembly.

### Evaluation of PathoScope 2.0 on simulated and real-data examples

We rigorously evaluated PathoScope 2.0 using two datasets. The first dataset consisted of in silico genomic sequencing reads simulated from 25 strains of bacteria that are commonly found in humans which includes five strains of *Escherichia coli*, five strains of *Staphylococcus aureus*, five strains of *Streptococcus pneumoniae*, and 10 other commonly occurring human bacterial strains. The second dataset contained clinical sequencing samples from the 2011 European outbreak of Shiga-toxigenic *E. coli* O104:H4. We demonstrate the improvements in PathoID 2.0 compared to PathoID version 1.0 in our simulation study of 25 bacterial strains. Using the clinical samples, PathoScope 2.0 is compared with other similar pipelines including PathoScope 1.0 for both accuracy of diagnosis and speed.

### Simulation study to evaluate improvements in PathoID 2.0

Our simulated data consisted of five sets of 100,000 simulated Illumina reads derived from each of the 25 strains of bacteria (see Methods). First, we processed the reads for each of the 25 bacterial strains individually using both PathoID version 1.0 and 2.0 with default parameter values and also PathoID 2.0 using a highly informative prior (see Methods). Although PathoID 1.0 was able to estimate the correct proportions of reads at the species level (100% to the particular species) for each of these 25 samples, it was not able to estimate the correct proportions of reads at the strain level (100% to the particular strain) for six samples (Additional file 2). In contrast, PathoID 2.0 using default parameters estimated the correct proportions of reads at the strain level (100% to the particular strain) for all the 25 samples. PathoID 2.0 with an informative prior estimated the correct proportions of reads at the strain level (100% to the particular strain) for 24 of the 25 samples, but was unable to estimate the correct proportion for one sample with *S. aureus* N315. The results are consistent for all five sets of simulated samples.

We also combined all the reads from the 25 strains, to create a dataset with all bacteria in equal proportions (expected proportion for each strain: 4%). Again, we applied both PathoID version 1.0 and 2.0 with default parameter values and added multiple informative priors (low, moderate, high; see Methods). We saw marked improvement in PathoID version 2.0 over version 1.0, including increased accuracy with informative priors (Table 1). For the 10 bacterial species that only included one strain per species, all methods performed well and estimated the correct read proportions. For the three species that contained multiple strains (*E. coli*, *S. aureus*, and *S. pneumoniae*), PathoID 1.0 and 2.0 at default parameters were able to identify all of the strains present, but struggled to estimate the correct read proportions. This failure demonstrates the tendency of the PathoID algorithm (at default parameters) to identify a single strain for each species, and if multiple strains or substrains are present it may reassign too many of the reads to a single strain. This tendency is an advantage in cases where there is only one strain of each species in the sample, but it leads to inaccuracies in the proportion estimates when multiple strains or substrains of the same species are present in the sample. The result of PathoID 2.0 with a highly informative prior matched closely with the expected proportions for 24 of the 25 strains, including 14 of the 15 cases with multiple strains of the same species. This demonstrates the value of using a highly informative prior when there are multiple strains of the same species in the sample, but we note that this comes at reduced effectiveness when there is only a single strain of each species in the sample. Finally, we repeated our simulation study using multiple mixtures of the 25 strains at random proportions, but the results were consistent with the mixtures at equal proportions, that is, the strains for which the proportions were not accurate with the mixtures at equal proportions matched with that of the mixtures at random proportions. Hence, we do not discuss them in detail here (see Additional file 2).

**Table 1:** Simulation study results

	**PathoID 1.0**	**PathoID 2.0**		
**Organism**	**Default**	**Default**	**ThetaPrior (Low)**	**ThetaPrior (High)**
*Bacteroides fragilis* 638R	4.00%	3.99%	3.99%	3.99%
*Bifidobacterium bifidum* BGN4	4.00%	3.99%	3.99%	3.99%
*Clostridium perfringens* ATCC 13124	3.99%	3.99%	3.99%	3.99%
*Enterococcus faecalis* V583	3.98%	3.99%	3.99%	4.00%
*Escherichia coli* 042	17.15%	4.01%	4.10%	4.02%
*Escherichia coli* 55989	0.57%	0.50%	1.51%	3.83%
*Escherichia coli* SE11	0.28%	10.10%	7.07%	3.74%
*Escherichia coli* SE15	0.71%	3.43%	3.73%	3.82%
*Escherichia coli* UMNK88	1.29%	1.95%	3.59%	4.16%
*Haemophilus influenzae* 10810	3.98%	3.99%	3.99%	3.99%
*Neisseria meningitidis* MC58	3.25%	3.99%	3.99%	3.92%
*Pseudomonas aeruginosa* DK2	4.00%	3.99%	3.99%	3.99%
*Staphylococcus epidermidis* ATCC 12228	3.97%	3.98%	3.98%	3.98%
*Streptococcus mitis* B6	2.94%	3.82%	3.87%	3.94%
*Streptococcus mutans* UA159	3.58%	3.99%	3.99%	3.99%
*Stapylococcus aureus* HO 5096 0412	0.31%	1.76%	2.48%	3.80%
*Stapylococcus aureus* JKD6008	0.05%	15.82%	13.40%	3.79%
*Stapylococcus aureus* MRSA252	0.69%	1.46%	2.14%	3.85%
*Stapylococcus aureus* N315	0.00%	0.68%	1.46%	1.01%
*Stapylococcus aureus* Newman	0.15%	0.33%	0.56%	3.48%
Streptococcus pneumoniae 670-6B	0.92%	3.28%	12.87%	4.43%
Streptococcus pneumoniae ATCC 700669	0.29%	0.99%	2.10%	4.23%
*Streptococcus pneumoniae* G54	0.16%	0.44%	1.17%	3.35%
*Streptococcus pneumoniae* Hungary 19A-6	19.42%	14.75%	2.58%	4.33%
*Streptococcus pneumoniae* Taiwan1 9 F-14	0.00%	0.66%	1.34%	2.83%

Comparison of PathoID 1.0 to 2.0 using simulated data (see the section titled ‘Simulation study to evaluate improvements in PathoID 2.0’ for details).

The one strain that was not estimated well using a highly informative prior was *S. aureus* N315, which had a final average read assignment percentage of 1.01% (Additional file 2). PathoID 2.0 with a highly informative prior incorrectly assigned the following average read percentages to neighboring strains: Mu50: 0.8%, Mu3: 0.7%, TW20, JH1, and JH9: 0.4%, 04-02981 and T0131: 0.3%, ST228: 0.2%, USA300 TCH1516: 0.1% and USA300 FPR3757: 0.1% (See Additional file 2). After further evaluation, we observed that PathoID failed with this strain due to the ‘sequencing errors’ in our simulated reads that caused some of the N315 reads to align more closely to the related strains. This phenomenon limited the ability of PathoID to correctly estimate the correct read proportions for this strain.

### Evaluation and comparison on clinical sequencing samples

We selected samples from fecal specimens obtained from patients with diarrhea during the 2011 European outbreak of Shiga-toxigenic *Escherichia coli* (STEC) O104:H4 (NCBI accession: ERP001956). To demonstrate the flexibility of PathoLib with the *E. coli* data, we constructed a targeted library containing only *E. coli* subspecies (taxID: 562). We note that many sequences contained in this library are redundant, and include separate sequence entries for complete genomes, individual chromosomes or plasmids, and distinct transcripts. This sequence redundancy allows PathoLib to identify the complete genome or chromosome for the strain if present, and also allows for the identification of the plasmid or genes present in the case of a horizontal transfer.

The updates to the PathoID algorithm increased the number of reads that were correctly assigned to the source genome, reduced the number of false positive genomes identified, and allowed for improved identification of multiple substrains in the same sample (Table 2). In addition, PathoScope 2.0 outperformed competing methods, such as RINS [12] and ReadScan [15], in computational efficiency, detection accuracy in terms of number of reads assigned to and overall ranking of STEC genomes, as well as in the interpretability of the results (Table 2). More detailed results from PathoScope (versions 1.0 and 2.0) and comparisons with two other near-complete pipeline methods, RINS and ReadScan, are detailed below.

**Table 2:** Comparison of PathoScope 2.0 against other methods with ELISA positive fecal samples

	**STEC Genome/Plasmid Rank (Plasmid Rank)**						**Computation time (h:min)**			
**Sample ID**	**PathoScope 2.0**		**PathoScope 1.0**		**ReadScan**	**RINS**	**PathoScope 2.0**	**PathoScope 1.0**	**ReadScan**	**RINS**
	**Rank**	**Proportion**	**Rank**	**Proportion**	**Rank (Plasmid)**	**Rank**				
2535.1^a^	1	39.4%	1	40.3%	9 (1)	8	8:29	7:21	16:46	1:27
2535.2	1	91.4%	1	87.8%	8 (1)	13	0:19	0:14	0:27	22:43
2535b^a^	1	36.9%	1	37.6%	13 (1)	36	2:47	2:25	7:26	1:26
2638	1	99.8%	1	95.7%	5 (1)	38	1:17	1:06	1:11	19:25
2661	1	95.7%	1	91.4%	8 (1)	7	0:36	0:32	1:58	8:57
2668	1	73.5%	1	85.3%	10 (1)	DNF	0:01	0:01	0:29:15	>24:00
2723^b^	1	99.8%	1	94.1%	5.5 (1)	37	0:14	0:12	0:43	15:28
2741	4	2.9%	28	0.0%	26 (1)	DNF	0:48	0:38	1:21:08	>24:00
2752	1	65.5%	2	10.1%	13 (1)	20	0:09	0:08	0:49	18:20
2758	1	99.7%	1	93.8%	8 (1)	38	0:16	0:15	0:52	10:13
2764	1	99.4%	1	96.6%	7 (1)	6	0:15	0:14	1:06	5:47
2772	1	99.6%	1	94.0%	4 (1)	8	0:00	0:00	0:16	12:22
2828	2	26.5%	2	5.3%	16 (1)	36	0:18	0:15	0:52	4:18
2840	1	99.5%	1	94.2%	8 (1)	14	1:52	1:40	1:58	19:08
2848	3	2.1%	6	0.7%	35 (3)	DNF	0:14	0:11	0:48	>24:00
2849	1	99.7%	1	95.2%	8 (1)	27	0:28	0:24	1:12:40	2:16:27
2878	1	99.4%	1	93.6%	8 (1)	DNF	0:07	0:06	1:04	>24:00
2880	1	87.9%	1	90.5%	11 (1)	37	0:04	0:04	0:26:33	2:35:38
2896	1	99.9%	1	95.2%	3 (1)	14	0:43	0:34	1:36:33	10:51:35
2971	1	99.9%	1	93.3%	8 (1)	38	0:23	0:19	0:56:59	5:05:27
3014	1	99.7%	1	92.3%	7 (1)	DNF	0:34	0:31	1:07:34	>24:00
3093	1	100.0%	1	88.7%	7 (1)	20	0:00	0:00	0:21:46	0:25:38
3132	1	96.5%	1	89.8%	15 (1)	11	0:07	0:06	1:36:49	8:56:37
3134	2	28.0%	2	5.8%	26 (1)	38	0:19	0:16	0:48:50	2:00:25
3135	1	99.7%	1	93.2%	8 (1)	DNF	0:12	0:10	0:58:49	>24:00
3185	1	91.4%	1	89.4%	9 (2)	6	0:22	0:19	0:51:03	17:34:13
3303	1	99.4%	1	94.2%	8 (1)	37	0:13	0:10	0:42:17	0:27:51
3549	8	0.7%	12	0.4%	65 (8)	9	0:11	0:09	0:41:51	20:41:23
3587	2	5.8%	3	2.5%	20 (7)	DNF	0:10	0:09	1:04:31	>24:00
3646	17	0.0%	5	1.2%	57 (19)	20	0:07	0:05	1:11:30	0:43:05
3751	2	5.8%	2	2.9%	19 (3)	DNF	0:22	0:19	1:11:05	>24:00
3852	1	100.0%	1	92.4%	5 (1)	13	0:00	0:00	0:18:15	1:08:41
3958	3	2.9%	5	1.1%	64 (7)	9	0:05	0:05	0:41:53	10:33:13
4112	7	0.7%	3	2.8%	50 (6)	38	0:08	0:08	0:41:39	13:05:36
4141	1	74.7%	1	83.5%	28 (2)	NI	0:03	0:03	1:01:14	10:57:57
4168	1	100.0%	1	95.2%	2 (1)	13	0:00	0:00	0:08:43	6:29:11
4198	2	1.4%	3	0.8%	36 (11)	37	0:43	0:33	1:20:30	12:17:53
4328^b^	1.5	21.7%	1	87.0%	7.5 (2)	DNF	0:05	0:05	0:56	>24:00
4508	3	6.5%	3	2.2%	37 (8)	38	0:05	0:04	0:44:12	14:44:41
5066	17	0.1%	13	0.2%	14 (1)	DNF	0:06	0:05	0:26:53	>24:00

^a^Used different parameters for bowtie2 alignment: ‘--very-sensitive-local --score-min L,280,0.0 -k 10’.

^b^There are multiple sequencing runs for this sample, so the results were averaged across replicates.

DNF: the algorithm did not finish in less than 24 h on a 16 cpu compute node; NI: the STEC O104:H4 genome was not identified by the method.

These are the results from the *O104:H4* study - Positive Samples. See the section titled ‘Evaluation and comparison on clinical sequencing samples’ for details.

When comparing PathoID versions 1.0 and 2.0, we observed that version 2.0 assigned more reads to the STEC genome in 31 of the 40 STEC positive samples. In these 40 samples, we observed mild to moderate increases in proportion of reads correctly reassigned, as well as reductions in number of incorrect genomes that were assigned reads. For example, for one of the samples (sample 3852), version 1.0 of the algorithm reassigned 92.4% of the reads to the correct O104:H4 substrain. However, four other incorrect *E. coli* strains each received between 1% and 2% of the reads. In contrast, with the updated scoring approach, 99.99% of the reads were correctly reassigned to the proper substrain. In addition, the original scoring approach assigned read proportions above 0.2% (range: 0.2% to 2.2%) to seven different incorrect *E. coli* strains; whereas with the new scoring system, the maximum incorrect read proportion was approximately 20 to 200 times smaller at 0.01%. The other 30 samples showed similar improvements (that is, shorter genome lists and more reads assigned to the STEC genome) using the updated scoring system.

In addition, we considered one sample in more detail (sample 2535) because this sample contained a mixture of distinct *E. coli* strains. Interestingly, PathoID found O104:H4 at 39.5% and a few other strains in high proportions as well including DH1: 12.0%, 55989: 11.9%, BL21(DE3): 11.8% and O127:H6 str. E2348/69: 8.4%. After processing this with a full bacterial reference library generated by PathoLIB, the sample still remained dominantly a mixture of *E. coli* strains, but PathoID did identify *Ruminococcus sp.* SR1/5 (6.9% of the reads) in the sample as well.

We compared the performance of PathoScope (versions 1.0 and 2.0), RINS, and ReadScan with these same STEC samples. The goal of the original study was to characterize STEC strains in a retrospective manner. In all 40 STEC positive samples, PathoScope (versions 1.0 and 2.0) identified the correct STEC genome with varying read assignment proportions (see Table 2). In 27 of the 40 samples, the STEC genome was the highest ranked genome, and in 35 samples the STEC genome was among the top three ranked genomes. In comparison, in all 40 samples ReadScan was able to identify the Shiga-toxin producing plasmid, ranking the correct plasmid first in 28 of the samples and in the top three hits for 33 samples. However, ReadScan could not discriminate between specific *E. coli* strains, indicating that ReadScan was very effective at identifying the pathogenic elements, but it could not identify the specific strains in which the plasmid resided because it does not connect plasmids, genes, or chromosomes from the same genome in the reference library. This reduces ReadScan’s ability to use unique plasmids and genes to identify the correct species/strains on the sample, particularly in cases where the pathogenic plasmid is transferred to a different bacterial strain or species. RINS was unable to accurately identify the correct STEC genome or plasmid in any of the samples. RINS employs a method of assembly of contigs from the reads and uses a local version of BLAST to classify contigs, which does not seem to work well when the reads are too small for assembly and when there are some read errors. RINS generated a large number of very small contigs that did not uniquely align to any specific genome with these samples. When the reads are too short and the coverage is not deep enough for assembly, as in this case, discriminatory information can be lost by an assembly approach because some of the reads that carry discriminatory information reads were not included in the contigs.

We also analyzed nine STEC negative samples from this dataset (Table 3). RINS did not score a STEC genome or plasmid very highly in any of the samples, but the results were very consistent (that is, genome/plasmid rankings) with the STEC positive samples. ReadScan ranked the STEC plasmid as one of the two highest scoring hits for four of the eight samples. Similarly, PathoID 1.0 also estimated the STEC genome proportions to be more than 1.0% in four of the samples. In contrast, PathoID 2.0 only estimated one of the samples to have a ‘high’ STEC percentage (1.4%). We note that these false positives were due to the fact that these reads were only aligned to the *E. coli* reference library, and when we used the more general bacterial reference library, we found that PathoID 2.0 estimated the STEC percentage as close to zero for all the samples.

**Table 3:** Comparison of PathoScope 2.0 against alternatives with ELISA negative fecal samples

	**STEC Genome/Plasmid Rank (Plasmid Rank)**						**Computation time (h:min)**			
**Sample ID**	**PathoScope 2.0**		**PathoScope 1.0**		**ReadScan**	**RINS**	**PathoScope 2.0**	**PathoScope 1.0**	**ReadScan**	**RINS**
	**Rank**	**Proportion**	**Rank**	**Proportion**	**Rank (Plasmid)**	**Rank**				
1253	3	1.4%	8	0.3%	33 (2)	38	0:03	0:03	0:41	18:10
4961	7	0.3%	6	0.6%	40 (11)	38	1:16	0:59	1:09:37	23:58:39
1122	14	0.0%	2	7.3%	24 (1)	38	0:00	0:00	0:21:43	
1196	NI	0.0%	2	3.0%	38 (1)	7	0:13	0:11	2:45:37	4:19:29
4096	25	0.0%	8	0.6%	70 (6)	14	0:07	0:06	0:29:09	0:06:00
1196b	4	0.8%	3	3.1%	31 (29)	9	0:04	0:04	1:27:27	7:03:26
4961b	8	0.2%	4	0.5%	35 (16)	39	0:15	0:12	0:30:49	21:30:20
1122b	15	0.0%	2	6.6%	26 (2)	38	0:03	0:02	0:17:41	5:32:59
4096b	22	0.0%	8	0.4%	76 (7)	36	0:01	0:01	0:14:37	0:18:45

NI: the STEC O104:H4 genome was not identified by the method.

These are the results from the *O104:H4* study - Negative Samples. See the section titled ‘Evaluation and comparison on clinical sequencing samples’ for details.

In addition to being more accurate than the other methods, PathoScope also outperformed RINS and ReadScan in terms of the computation time needed to complete the analysis. On a cluster node with 16 CPUs and 256 GB of RAM, PathoScope 2.0 required an average of 17 min of compute time, whereas ReadScan required an average of 50 min and RINS required an average of more than 8 h for the 30 completed samples (10 of the samples did not finish in less than 24 h). PathoID version 1.0 was on average 3 min faster than PathoID version 2.0, but this computational time increase is almost entirely due to added features in version 2.0, such as better genome annotation, detailed result reporting, and interaction with other PathoScope modules. We also repeated the experiment with a larger target database containing all bacterial sequences. Only PathoScope 2.0 was able to finish this run successfully in a reasonable computational timeframe (<24 h per sample).

## Conclusions

PathoScope 2.0 provides a complete modular bioinformatics workflow to analyze metagenomic sequence data from clinical or environmental samples. The pipeline helps researchers to efficiently generate custom reference libraries, align reads to a target library, filter host reads, overcome read alignment ambiguity, characterize target diversity, and annotate results. Our simulated and real-data examples show that PathoScope 2.0 is a highly sensitive and efficient approach for metagenomic analysis, without the need for computationally intensive database preprocessing and time-consuming *de novo* assembly. PathoScope 2.0 is a fast and modularized pipeline for which we provide a comprehensive command line interaction so that more advanced users can selectively run parts of the modules, but is user-friendly enough to be used by researchers with weaker computational backgrounds.

The libraries generated by PathoLib contain all available reference genome sequences (for example, NCBI nucleotide) that meet the user-defined genome or taxID selection set. These sequences are often redundant, and may include separate entries for the complete genome sequences, individual chromosomes or plasmids, and distinct transcript sequences. Thus, for any given bacterial genome, chromosome, or plasmid, the reference library will likely contain all three elements in separate forms. PathoScope will then use the reads to identify the most likely source for the reads. For example, if the reads only align to the plasmid, then PathoScope will select the plasmid as the source. If the reads align to both the plasmid and the surrounding sequence and/or neighboring chromosomes, the entire genome will likely be selected. In the *E. coli* example above, this allowed PathoScope to identify the entire STEC genome of interest, whereas ReadScan was only able to identify the STEC plasmid.

One possible limitation to the reference-based approach used by PathoScope is that it relies on the genome for each strain to be present in the library in order to achieve a precise identification. We note that PCR and microarray based approaches fail when the target is unknown as well, as we cannot make PCR primers when we do not know the sequence. In cases where the species is truly novel with no closely related sequenced genomes, PathoScope will not be able to identify the species/strain of origin for the reads. However, we have previously shown that when the specific strain is not in the reference, PathoID can successfully identify the nearest species/strain in the library [16]. For example, we showed that when the *E. coli* O104:H4 genome was not in the library, PathoID successfully identified the 55989 strain, which has been previously established as the nearest sequenced strain to O104:H4 [16]. We observed a similar effect when we removed the correct *F. tularensis* strain from the library [16]. Therefore, based on these examples, we have demonstrated that PathoScope is a robust and useful tool for the majority of the metagenomic studies. Furthermore, the modular framework of PathoScope 2.0 allows for additional assembly-based modules to be added to the framework that would be able to identify completely novel genomes.

## Methods

### Improvements to the PathoID reassignment model

The PathoID module essentially comprises our previously published PathoScope version 1.0 software [16]. PathoID inputs aligned sequencing reads in SAM format [18], and returns an updated alignment file of read reassignments (also SAM format) along with summary report containing read proportions assigned to each genome in the reference library. The modular PathoScope 2.0 framework introduces improvements in the original PathoID algorithm, as well as several novel functionalities that extend PathoID into a complete workflow for sequence-based metagenomic profiling. These include: (1) the automatic extraction of custom reference genome libraries (PathoLib); (2) the construction of reference indices (splitting large libraries into separate indices, if necessary), the alignment of reads to the target libraries, and the filtering reads that align to the host or other filter libraries (PathoMap); (3) the preprocessing of reads with complete and parallel quality control (PathoQC); (4) the annotation of all sequences in the reference library (PathoDB); and (5) detailed reports on organisms, reads, genes, and gene products identified in the study (PathoReport).

PathoID utilizes a missing data mixture modeling approach, where the template genome of origin is the ‘missing data’. It integrates information from the read alignment with information obtained by borrowing strength across all reads from the sample to reassign ambiguous reads to the most likely source genome in the library. The PathoID likelihood contains a parameter that penalizes reads that align to multiple genomes; thus, increasing the impact of uniquely mapping reads on the reassignment result. Conversely, the previously published PathoID algorithm does not penalize reads relative to their ‘best’ read alignment, meaning that reads that align perfectly have the same influence on the result as reads whose best alignment contains base mismatches. Furthermore, in the PathoID version 1.0, reference genome length is also not expressly modeled in the likelihood, yielding an advantage for completed genomes over smaller incomplete sequence fragments. In cases where the closest reference genome is incompletely assembled, PathoID might tend to identify a more genetically distant completed genome. Finally, although PathoID was initially developed under a Bayesian framework, the PathoID version 1.0 software does not allow users to easily modify the preset priors. This becomes extremely important in cases where there is more than one substrain of the same species present in the same sample. To accommodate these concerns, we introduced the following changes into version 2.0 of the PathoID reassignment model:

### Read alignment score

To increase the influence of perfect match reads in PathoID, we implemented a weighted likelihood based approach, which consists of weighting the reads in the log-likelihood based on their relative alignment likelihood (exponentiated alignment ‘bit score’). A more general form of this weighted likelihood formulation has been rigorously evaluated and shown to share the same asymptotic features of the genuine likelihood function [19]. In the finite sample metagenomic context, the reads with a higher alignment scores will have more influence on the results than reads that align with more mismatches.

### Reference genome length

We weight likelihood contributions for each alignment by the inverse of the length of a target genome. Similar approaches for adjusting for length in sequence alignments are well established, for example, BLAST ‘E-value’ [20].

### Read alignment scores

For most SAM alignment files (including Bowtie 2 alignments), our original read assignment algorithm used the SAM-MAPQ score to provide relative alignment probabilities. We now use the read alignment bit score (AS) from the Bowtie2 output. The AS is also standardized by adding the read length and normalizing the score to a fixed range so that the likelihood calculations of the EM algorithm do not exceed upper or lower computational precision limits.

### Flexible user-defined prior values

The PathoID reassignment module was developed under a Bayesian framework that allows researchers to insert prior information into the genome identification approach. This provides an option for the user to ‘bias’ the PathoID reassignment in cases where the researcher has a priori knowledge of the likely content of the metagenomic sample. In addition, by assigning a prior value on the read ambiguity penalty, the user can modify the severity of the penalty placed on ambiguous reads. This becomes extremely important in cases where there are multiple substrains of the same species in the sample. With non-informative priors in this penalty parameter (default in version 1.0), PathoID has the tendency to search for a ‘parsimonious’ solution, often at the cost of assigning all non-unique reads to only one of the highly similar substrains present in the sample. By adding an informative prior to the penalty parameter, the PathoID reassignment will be less precise in its read assignments, but will more closely estimate the correct proportions assigned to the multiple substrains - usually at the cost of decreased accuracy of the unique strains in the sample. In version 2.0 of the PathoID algorithm, we allow the user to modify the prior values placed on the genome proportion and read penalty parameters. To determine whether an informative prior is needed, we suggest that the user considers an alignment to a core genome region for species that might have multiple strains present in the sample. If there are multiple closely related strains present in the sample then it is best to use highly informative prior value. On the contrary, if it appears that there is one particular strain to be identified, then it is best to use default or low informative prior value.

### Simulation study details

We generated a simulation study dataset to assess the performance of improvements made to PathoID in version 2.0 of the software. In particular, we wanted to evaluate the impact of informative prior information on the ability to accurately estimate genome proportions when multiple strains of the same species are present in the sample. To do this, we simulated sequencing reads from 25 strains of bacteria which includes five *Escherichia coli* strains (O42, 55989, SE11, SE15 UMNK88), five *Staphylococcus aureus* strains (JKD6008, Newman, MRSA252, HO 5096 0412, N315), five *Streptococcus pneumoniae* strains (670, ATCC_700669, G54, Hungary19A, Taiwan19F) and 10 other common bacterial strains (*Bacteroides fragilis* 638R, *Bifidobacterium bifidum* BGN4, *Clostridium perfringens* ATCC 13124, *Enterococcus faecalis* V583, *Haemophilus influenzae* 10810, *Neisseria meningitidis* MC58, *Pseudomonas aeruginosa* DK2, *Staphylococcus epidermidis* ATCC 12228, *Streptococcus mitis* B6, *Streptococcus mutans* UA159). The phylogenetic relationships between these strains and other strains available in the NCBI database are given in Additional file 3. We used the Mason read simulator [21] to generate five sets of 100,000 reads for each strain simulating 100 bp single-end sequencing reads using an ‘Illumina-like’ sequencing error model; Mason parameters: ‘illumina -s ## -N 100000 -sq -n 100 -i -hs 0.0 -hi 0 -hnN -nN’ (-s (Seeds) = 1101, 1102, 1103, 1104, 1105). We used PathoLib (-t 2 --subTax) to generate a reference library containing all bacteria. We used PathoMap (default parameters) to index and align the reads to the bacterial library. PathoMap automatically splits the bacterial library into smaller parts (<4.3 GB in size) that the Bowtie2 aligner can process and combines the alignment files together for the final results. We then applied PathoID (versions 1.0 and 2.0) to the simulated datasets. PathoID version 2.0 was applied with default parameters and with three informative priors (low, moderate, high). The low informative prior corresponds to ‘-thetaPrior 1000’, moderate informative prior corresponds to ‘-thetaPrior 50000’ and high informative prior corresponds to ‘-thetaPrior 10**88’. The thetaPrior value represents the number of non-unique reads that are not subject to reassignment.

### European *E. coli* outbreak samples

We obtained data from the 2011 European outbreak of Shiga-toxigenic *Escherichia coli* O104:H4 [22]. The dataset consisted of 150 bp paired-end sequencing reads from fecal samples obtained from patients impacted by outbreak (NCBI accession number: ERP001956). We used reads from 21 sequencing runs originating from 19 samples for which a standard enzyme-linked immunosorbent assay (ELISA) identified a bacterial shiga toxin. The data were pre-filtered for human nucleic or mitochondrial reads and only included reads that have a ‘best-hit’ alignment to bacterial taxon genomes [22]. We applied PathoScope 2.0, PathoScope 1.0, ReadScan, and RINS to the datasets using the parameters given in Additional file 4. Because of the nature of this study and to demonstrate the flexibility of PathoLib, we constructed a target library containing all *E. coli* subspecies (taxID: 562) in the NCBI RefSeq nucleotide database (download date: January, 2013). PathoLib extracted 46,640 sequence entries from a total 54 different *E. coli* strains. We used these samples to compare the improved performance of PathoScope 2.0 over PathoScope 1.0, and have also included comparisons with two other near-complete pipeline methods, RINS and ReadScan. We recorded the rank of the *E. coli* O104:H4 genome, the highest-ranking pathogenic *E. coli* plasmid (ReadScan only), the proportion of reads assigned to O104:H4 (PathoID only), and the total computational time required. We also limited computational time to 24 h on a 16 cpu node (24 × 16 = 384 cpu hours) for each individual sample.

### PathoScope 2.0 software tutorial

We provide a complete tutorial (Additional file 5) that covers the basics of using PathoScope to analyze metagenomic samples. The tutorial provides a step-by-step procedure to transform unintelligible sequencing reads into a meaningful picture of the microbial content present in a sample.

## Abbreviations

ELISA: Enzyme-linked immunosorbent assay; NCBI: National Center for Biotechnology Information; STEC: Shiga-toxigenic *Escherichia coli*; taxID: NCBI taxonomy identification.

## Competing interests

The authors declare that they have no competing interests.

## Authors’ contributions

CH: Conception and design, software development, STEC data analysis, manuscript writing, final approval of the manuscript. SM: Software design and development, analysis of simulation data, STEC data analysis, manuscript writing, final approval of the manuscript. YS: Evaluation of algorithms included in the workflow, establishment parameter presets, critical revision and final approval of the manuscript. JFPR: Evaluation of algorithms to be included in the workflow, construction of figures, critical revision and final approval of the manuscript. ALB: Generation of simulation dataset, evaluation of algorithms to be included in the workflow, construction of figures, critical revision and final approval of the manuscript. ECN: Designed and wrote the software tutorial, critical revision and final approval of the manuscript. KAC: Conception and design, financial support, critical revision and final approval of the manuscript. WEJ: Conception and design, financial support, analysis of simulation data, manuscript writing and final approval of the manuscript. All authors read and approved the final manuscript.

## Supplementary Material

Additional file 1 **PathoReport xml files for one sample (sample id: 4168) before and after PathoID.** The MAP_4168-H-STEC.xml file is the PathoReport generated from the sam alignment file before PathoID and updated_MAP_4168-H-STEC.xml is the PathoReport generated from the updated sam alignment file created by PathoID. The xml file contains more detailed results, including the reads assigned to each genome and contiguous sequences (contigs) constructed from overlapping reads. Click here for file

Additional file 2 **Simulation study results.** This file has the complete results from the simulation study with eight tabs at the bottom. There is a caption in each of the sheets at the bottom explaining the table present in each of the sheets. Click here for file

Additional file 3 **Phylogenetic relationships of the *S. aureus*, *E. coli*, *and S. peumoniae *** **genomes.** The genomes that are highlighted in red boxes were used for generating the reads. These images were obtained and modified from the NCBI: http://www.ncbi.nlm.nih.gov/genome/?term=staphylococcus%20aureus, http://www.ncbi.nlm.nih.gov/genome/?term=escherichia%20coli, http://www.ncbi.nlm.nih.gov/genome/?term=streptococcus%20pneumoniae. Click here for file

Additional file 4 **Parameter values used for the *E. coli *** **study unless explicitly stated otherwise for some cases (for one 2535 sample, a different PathoMap parameter was used, which is the following: ‘--very-sensitive-local --score-min L,280,0.0 -k 10’).** Click here for file

Additional file 5 **Pathoscope 2.0 user tutorial.** This tutorial demonstrates the basics of using PathoScope 2.0 to analyze metagenomic samples. We provide a step-by-step procedure that will transform your unintelligible sequencing reads into a meaningful picture of the microbial content present in a sample. Click here for file
