# Supplementary material for: PathoScope 2.0: a complete computational framework for strain identification in environmental or clinical sequencing samples
Source: Microbiome. 2014 Sep 5;2:33. doi: 10.1186/2049-2618-2-33 (PMC4164323; doi:10.1186/2049-2618-2-33)
Supplement: Additional file 3 — Phylogenetic relationships of the S. aureus , E. coli , and S. peumoniae genomes. The genomes that are highlighted in red boxes were used for generating the reads. These images were obtained and modified from the NCBI: http://www.ncbi.nlm.nih.gov/genome/?term=staphylococcus%20aureus, http://www.ncbi.nlm.nih.gov/genome/?term=escherichia%20coli, http://www.ncbi.nlm.nih.gov/genome/?term=streptococcus%20pneumoniae. [file 2049-2618-2-33-S3.pdf]

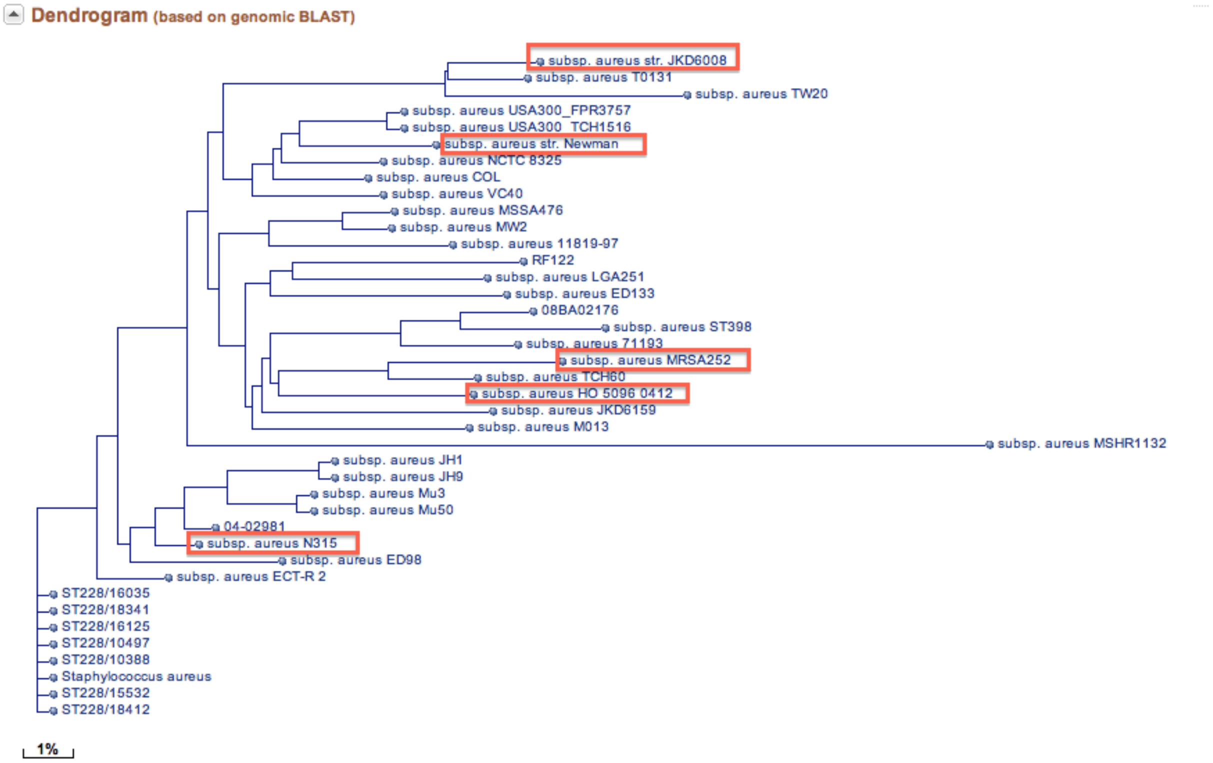

Supplementary Figure 1: Phylogenetic relationships of the *S. aureus* genomes used in the simulation study. The genomes that are highlighted in red boxes were used for generating the reads. This image was obtained and modified from the NCBI: http://www.ncbi.nlm.nih.gov/genome/?term=staphylococcus%20aureus.

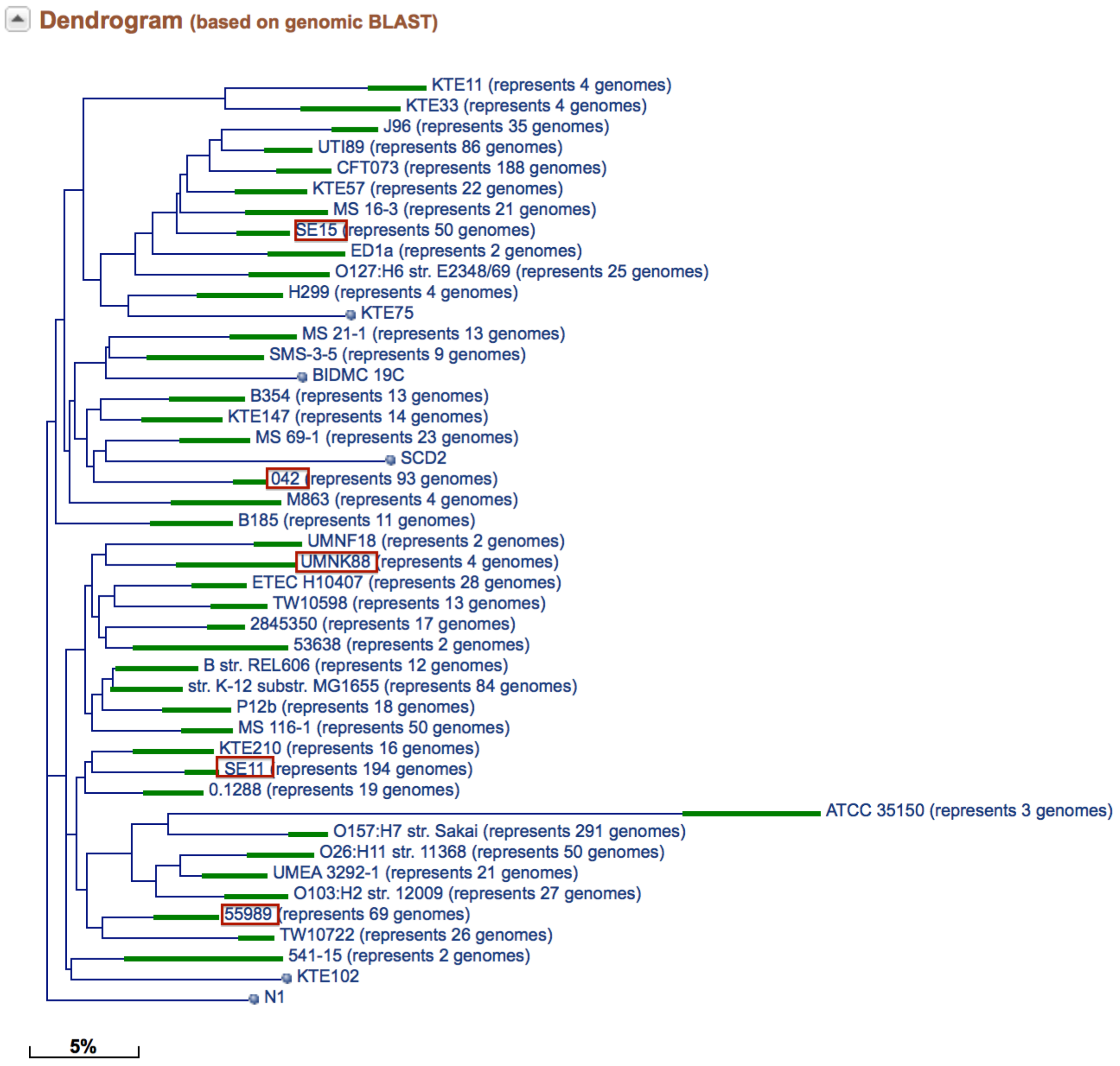

Supplementary Figure 2: Phylogenetic relationships of the *E. coli* genomes used in the simulation study. The genomes that are highlighted in red boxes were used for generating the reads. This image was obtained and modified from the NCBI: http://www.ncbi.nlm.nih.gov/genome/?term=escherichia%20coli

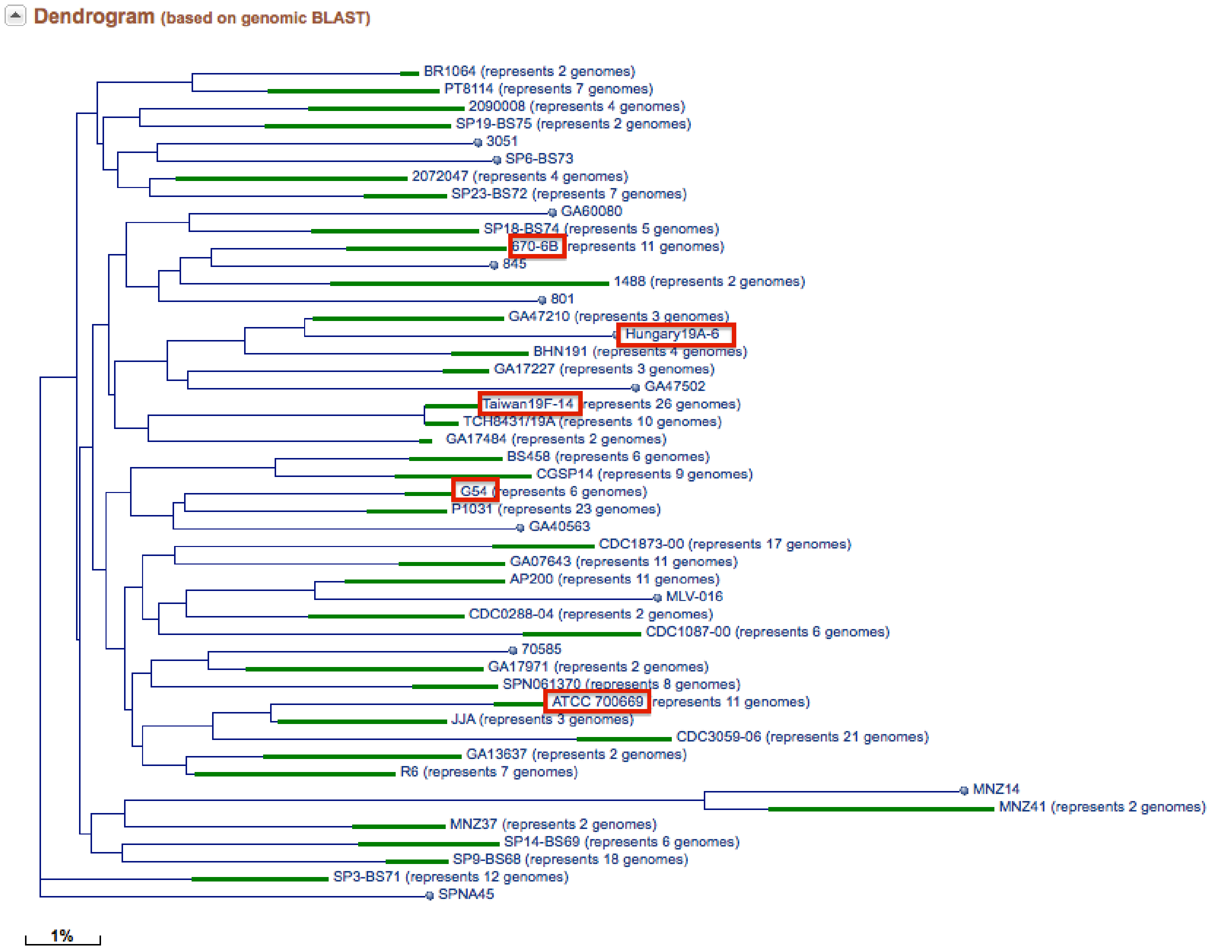

Supplementary Figure 3: Phylogenetic relationships of the *E. coli* genomes used in the simulation study. The genomes that are highlighted in red boxes were used for generating the reads. This image was obtained and modified from the NCBI: http://www.ncbi.nlm.nih.gov/genome/?term=streptococcus%20pneumoniae
