# Supplementary material for: PathoScope 2.0: a complete computational framework for strain identification in environmental or clinical sequencing samples
Source: Microbiome. 2014 Sep 5;2:33. doi: 10.1186/2049-2618-2-33 (PMC4164323; doi:10.1186/2049-2618-2-33)
Supplement: Additional file 4 — Parameter values used for the E. coli study unless explicitly stated otherwise for some cases (for one 2535 sample, a different PathoMap parameter was used, which is the following: ‘--very-sensitive-local --score-min L,280,0.0 -k 10’). [file 2049-2618-2-33-S4.pdf]

Supplementary Table 1: *Parameter values used for the E. coli study.*

Method	Parameter Settings
PathoScope 2.0	PathoLib: -taxonIds 562 --subTax<br>PathoMap: -targetAlignParams "--very-sensitive-local -k 500 --score-min L,205,0.0"<br>PathoID: default settings<br>PathoReport: --contig
PathoScope 1.0	default settings
ReadScan	index: -k 13 -s 6<br>smalt: -d -1 -y 0.94 -n 2 -f samsoft
RINS	bowtie_mismatch = 3<br>paired_fragment_length = 350<br>min_contig_length = 300<br>trinity_threads = 10<br>blastn_evalue_thrd = 0.05<br>similarity_thrd = 0.8<br>iteration = 2<br>raw_read_length = 150<br>chop_read_length = 25<br>minIdentity = 80<br>compress_ratio_thrd = 0.5
