# Supplementary material for: PathoScope 2.0: a complete computational framework for strain identification in environmental or clinical sequencing samples
Source: Microbiome. 2014 Sep 5;2:33. doi: 10.1186/2049-2618-2-33 (PMC4164323; doi:10.1186/2049-2618-2-33)
Supplement: Additional file 5 — Pathoscope 2.0 user tutorial. This tutorial demonstrates the basics of using PathoScope 2.0 to analyze metagenomic samples. We provide a step-by-step procedure that will transform your unintelligible sequencing reads into a meaningful picture of the microbial content present in a sample. [file 2049-2618-2-33-S5.pdf]

PathoScope 2.0 tutorial

February 2013. Version 0.01

Species identification and strain attribution with unassembled sequencing data

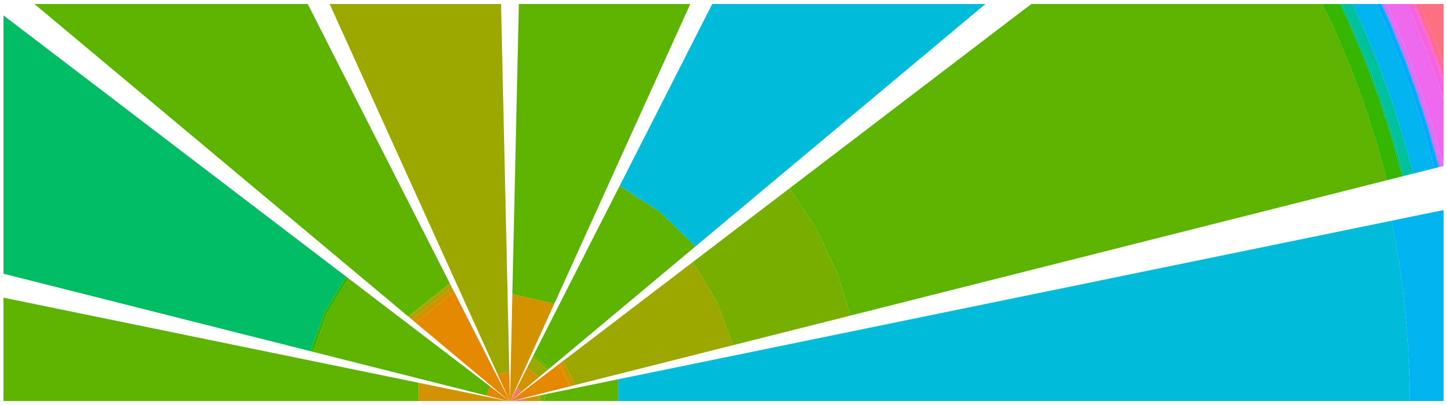

In this tutorial you will learn the basics of using PathoScope to analyze your metagenomic samples. We will provide you with a step-by-step procedure that will transform your unintelligible sequencing reads into a meaningful picture of the microbial content present in your sample.

PathoScope is developed at the Johnson Lab at Boston University

W. Evan Johnson, Ph.D.
Division of Computational Biomedicine
Boston University School of Medicine
72 E. Concord St., E-645
Boston, MA 02118

Developers:
Solaiappan Manimaran
Changjin Hong

For support queries, please open a ticket or contact us at pathoscope@users.sourceforge.net

PathoScope is distributed freely under the GNU General Public License and can be downloaded at:

http://sourceforge.net/projects/pathoscope/

Citation:
Francis, O. E., Bendall, M., Manimaran, S., Hong, C., Clement, N. L., Castro-Nallar, E., G. Bruce Schaalje, Mark J. Clement, Keith A. Crandall & Johnson, W. E. (2013). Pathoscope: Species identification and strain attribution with unassembled sequencing data. Genome research, 23(10), 1721-1729.

Table of Contents

The PathoScope Approach 3
Quick Start 4
Test PathoScope and Quick Example 4
Step-by-Step Example 5
1. Make sure you have all the ingredients 5
2. Let's build your library 6
2.1 Let' try using a MySQL database first (OPTIONAL)... 7
2.2 ...And now let's try without a MySQL database 7
2.3 How to setup a MySQL database 9
3. The PathoMap module 10
4. The PathoID module 12
5. The PathoReport module 14
6. Optional Modules 14
7. Run Times 14
References 15

The PathoScope approach

PathoScope is composed of three core and three optional modules that together provide a complete framework to assign unassembled sequencing reads to their genomes of origin (Diagram 1). The key difference of the PathoScope approach against others lies in its Bayesian read reassignment model (part of PathoID) that upweights uniquely mapped reads and uses them to guide the reassignment of ambiguosly mapped reads. For specific information about the model go to: *Francis et al. 2013* - http://genome.cshlp.org/content/23/10/1721.full

Diagram 1:

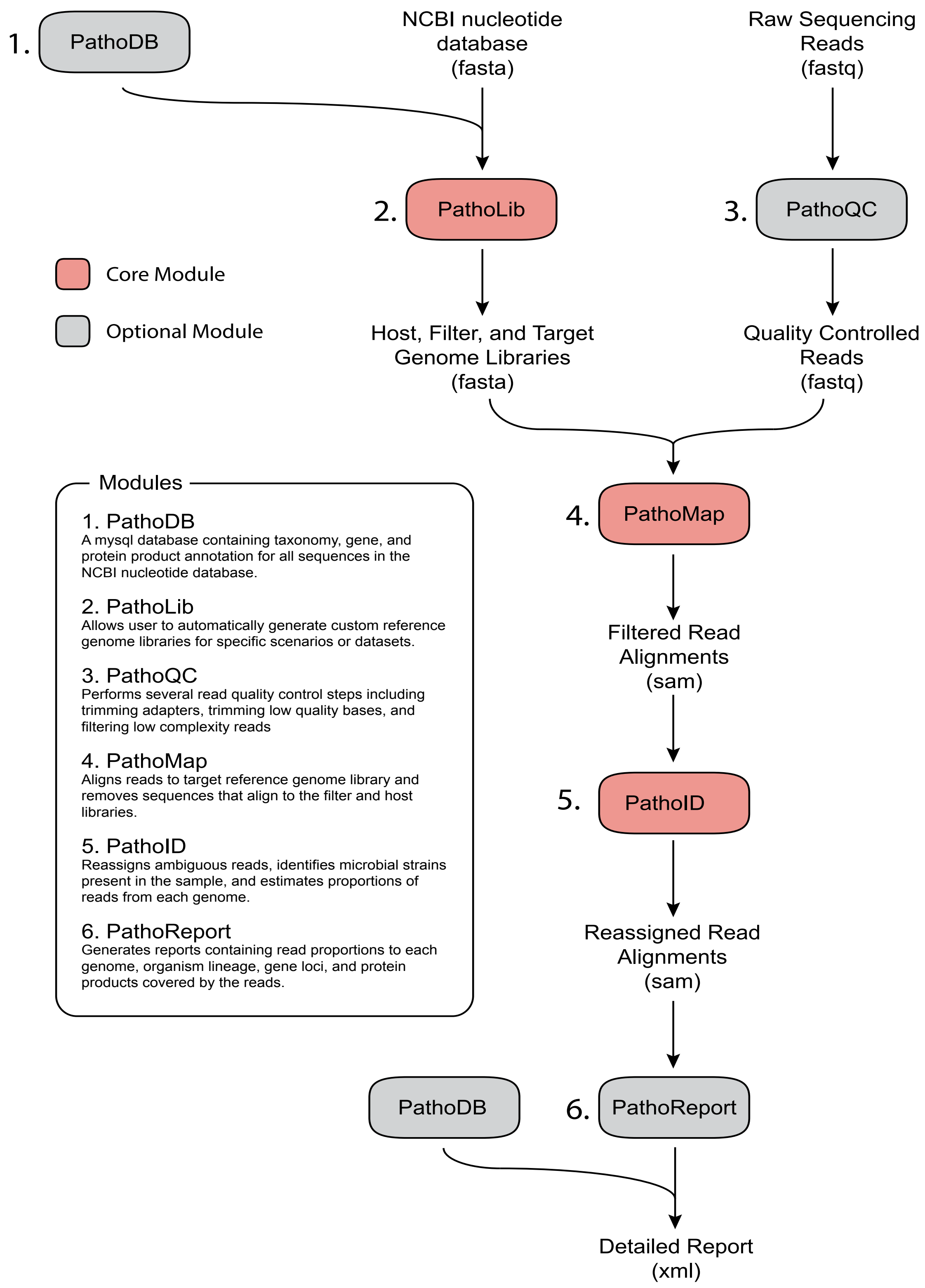

Quick Start

If you can't wait to start using PathoScope here are directions to get you started right away:

0. Make sure you have Python 2.7.3 or higher installed and added to your PATH variable. Alternatively, you could use an older version of Python provided that you install the argparse module (https://pypi.python.org/pypi/argparse)

1. Go to http://sourceforge.net/projects/pathoscope/ and download the latest version of PathoScope

2. Change directory to your download folder and extract the compressed file, e.g.,
   `$ tar xvf pathoscope_2.0.tar.gz`

3. Change directory to where you extracted the package and simply run:

```
$ python pathoscope/pathoscope.py -h          #General top level usage information
$ python pathoscope/pathoscope.py LIB -h      #Usage information for LIBrary module
$ python pathoscope/pathoscope.py MAP -h      #Usage information for MAPping module
$ python pathoscope/pathoscope.py ID -h       #Usage information for IDentification module
$ python pathoscope/pathoscope.py REP -h      #Usage information for REPort module
```

Test PathoScope and quick example

1. Change directory to `pathoscope/pathomap/bowtie2wrapper/unittest` and simply run `python testBowtie2Wrap.py`

2. Change directory to "pathoscope/pathoid/unittest" and simply run `python testPathoID.py`

There is a sample alignment file called 'MAP_3852_align.sam' that is included with this package in the example folder to test PathoID and PathoReport modules.

3. Generate TSV(Tab Separated Value) file Report that can be opened in Excel and an updated alignment file:

```
$ python pathoscope/pathoscope.py  ID -alignFile example/MAP_3852_align.sam -expTag 3852 -outDir re-
sults
```

4. Generate XML file and TSV file Report using the pathoReport module:

```
$ python pathoscope/pathoscope.py  REP -samfile results/updated_MAP_3852_align.sam -outDir results
```

Step-by-step example

1. Make sure you have all the ingredients

In this section, we will investigate the microbial content of Sequence Read Archive (SRA) run SRR167721. These data are derived from Lung swab / tissue from titi monkeys with pneumonia published by *Chen et al. (2011).* To obtain the proper fastq file, you can go to http://www.ncbi.nlm.nih.gov/sra/ and search for the SRA object SRR167721 as shown below:

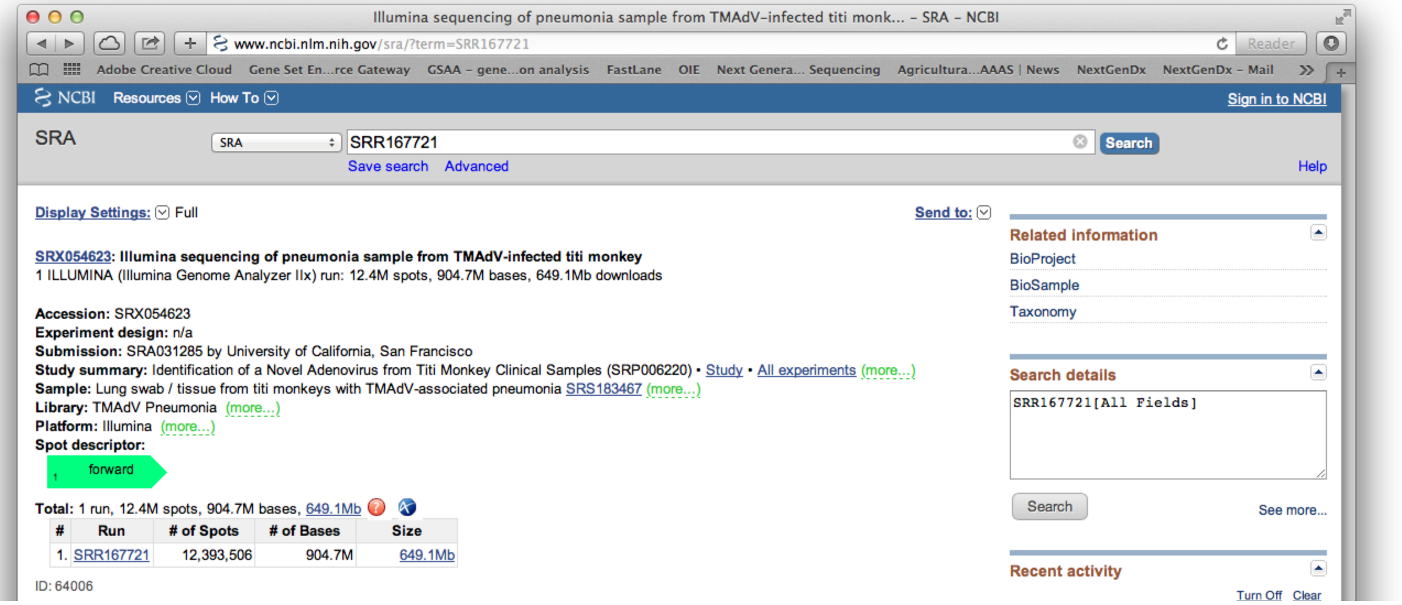

Then simply follow the on-screen directions to download the data. The SRA uses its own file format (.sra) so you need to convert the resulting file to .fastq. Please check the SRA toolkit documentation for information on how to get and install the appropriate programs to convert from sra to fastq (http://www.ncbi.nlm.nih.gov/books/NBK47540/). In particular, you could use the binary version of fastq-dump `$ fastq-dump SRR167721`

If you haven't downloaded PathoScope 2.0 yet, please do so at http://sourceforge.net/projects/pathoscope/ and extract the files by issuing something like this:

```
$ tar xvf pathoscope_2.0.tar.gz
```

PathoScope is designed to work on Linux-based machines like most computer clusters, all the flavors of Linux (e.g., Ubuntu, OpenSuse, RedHat, etc.), and Apple desktop and laptop machines. If you are a Windows user, it should work just with the standard python setup.

The only PathoScope dependency is Bowtie2; you need to make sure you have it in your $PATH.

You also need Python 2.7.3 or higher installed in your system and added to your PATH variable (usually by default in most systems). Check your Python version by issuing the following command from the terminal:

```
$ python -V
```

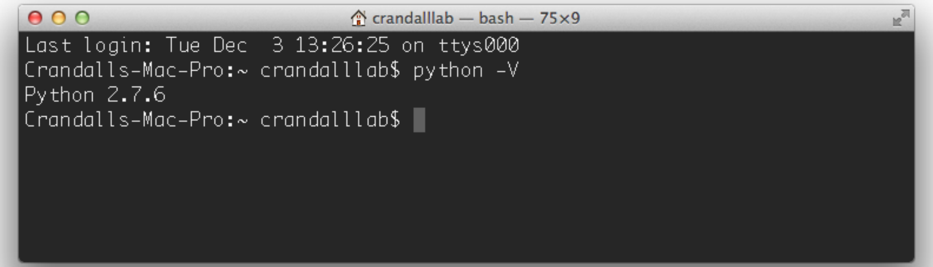

2. Let's build your library

PathoScope Library module (PathoLib) is designed to help you collect sequence data from NCBI that you deem pertinent for a given analysis. Basically, you need to think about your potential targets, i.e., what would you expect to find in your metagenomic sample that you want to identify, and your filters, i.e., what would you like to filter out from your data that are not part of the target analysis. Typically, you would want to look for any microbes (virus, bacteria, fungi) and discard any host reads and artificially added sequences like PhiX174.

So let's get some target sequences for our analysis:

°Change directory (cd) to PathoScope folder
°Create a directory to contain your library data
°cd into pathoscope directory and call PathoLib module help for details

```
$ cd Desktop/pathoscope2/
$ mkdir library
$ cd pathoscope
$ python pathoscope.py LIB -h
```

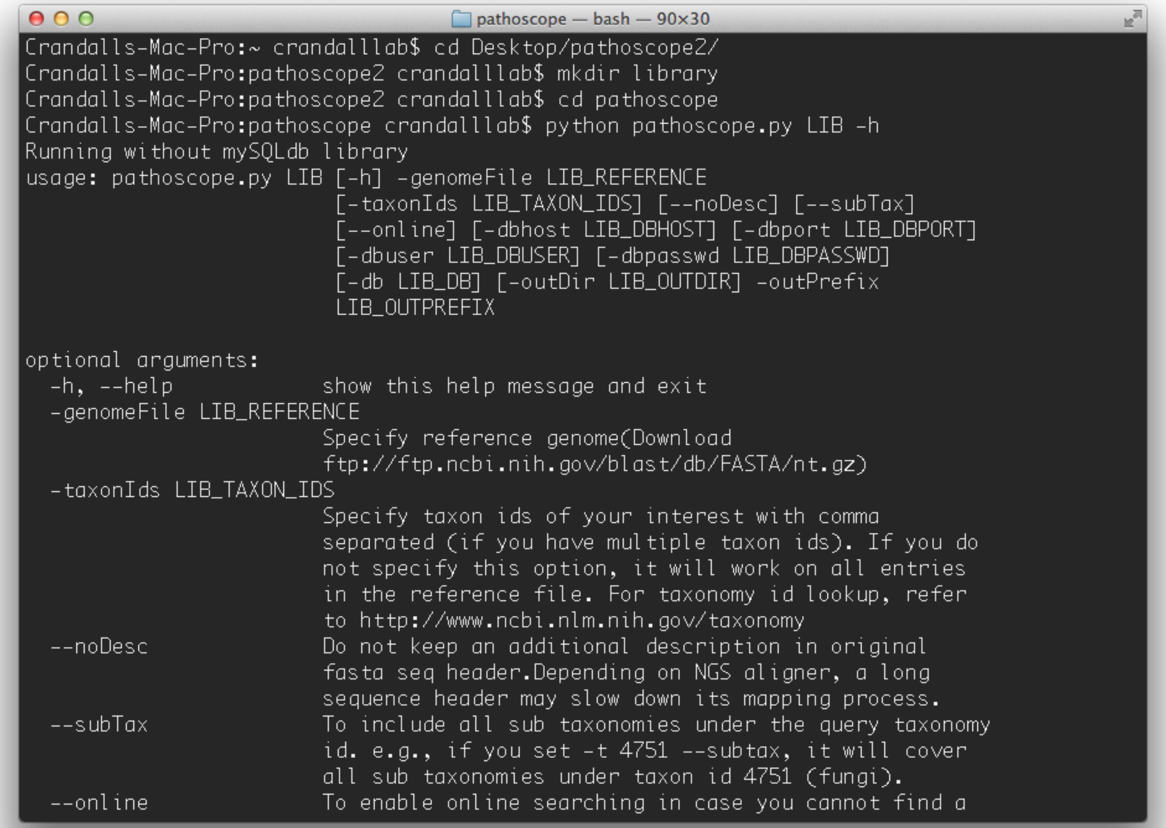

```
pathoscope — bash — 90×30
Crandalls-Mac-Pro:~ crandalllab$ cd Desktop/pathoscope2/
Crandalls-Mac-Pro:pathoscope2 crandalllab$ mkdir library
Crandalls-Mac-Pro:pathoscope2 crandalllab$ cd pathoscope
Crandalls-Mac-Pro:pathoscope crandalllab$ python pathoscope.py LIB -h
Running without mySQLdb library
usage: pathoscope.py LIB [-h] -genomeFile LIB_REFERENCE
                         [-taxonIds LIB_TAXON_IDS] [--noDesc] [--subTax]
                         [--online] [-dbhost LIB_DBHOST] [-dbport LIB_DBPORT]
                         [-dbuser LIB_DBUSER] [-dbpasswd LIB_DBPASSWD]
                         [-db LIB_DB] [-outDir LIB_OUTDIR] -outPrefix
                         LIB_OUTPREFIX

optional arguments:
  -h, --help            show this help message and exit
  -genomeFile LIB_REFERENCE
                        Specify reference genome(Download
                        ftp://ftp.ncbi.nih.gov/blast/db/FASTA/nt.gz)
  -taxonIds LIB_TAXON_IDS
                        Specify taxon ids of your interest with comma
                        separated (if you have multiple taxon ids). If you do
                        not specify this option, it will work on all entries
                        in the reference file. For taxonomy id lookup, refer
                        to http://www.ncbi.nlm.nih.gov/taxonomy
  --noDesc              Do not keep an additional description in original
                        fasta seq header.Depending on NGS aligner, a long
                        sequence header may slow down its mapping process.
  --subTax              To include all sub taxonomies under the query taxonomy
                        id. e.g., if you set -t 4751 --subtax, it will cover
                        all sub taxonomies under taxon id 4751 (fungi).
  --online              To enable online searching in case you cannot find a
```

You have two ways of running PathoLib. 1) You could set up or use an existing MySQL database from where to draw your library or 2) you can download NCBI's nucleotide database (10 GB) from their ftp site (ftp://ftp.ncbi.nih.gov/blast/db/FASTA/nt.gz) and get your data from there. Either way you'll end up with files representing your *Filter* or *Target* libraries.

°Within pathoscope2 directory, create a directory to contain your data files (name it `data`)
°cd to it and download the latest nucleotide database from NCBI
°Decompress

```
$ mkdir data
$ cd data
$ wget ftp://ftp.ncbi.nih.gov/blast/db/FASTA/nt.gz
$ gunzip -dc nt.gz > nt.fasta
```

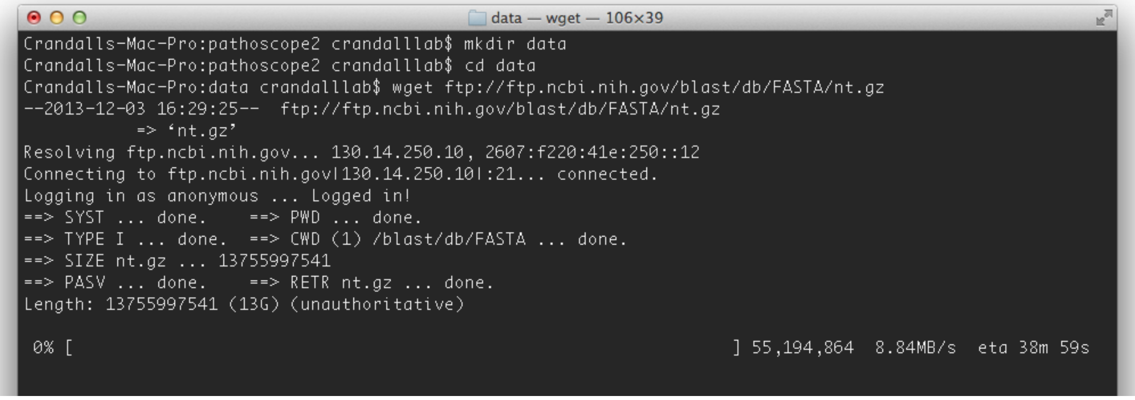

Now, you should have a file named nt.fasta within the pathoscope2/data directory. Our next step is collecting sequence data that matches viruses (Taxonomy ID 10239) so that we create a *target library*.

2.1 Let's try using a MySQL database first (OPTIONAL)...

For this option, you will need to have access to a MySQL database containing sequence data information, and the python package MySQLdb (http://sourceforge.net/projects/mysql-python/). Also, make sure you have `MySQLdb` in your PYTHONPATH.
Then, you should issue something like this:

```
$ python pathoscope.py LIB -genomeFile ../data/nt.fasta -taxonIds 10239
--subTax -dbhost localhost -dbuser user -dbpasswd xxxxx -outPrefix virus_
sql
```

Where -dbhost, -dbuser, and -dbpasswd are your credentials to access the SQL database. In the backend, PathoLib is going to take the file nt.fasta, grab GI numbers, query your SQL database, and retrieve taxonomy information to be prepended to your resulting file. Your resulting file will have all NCBI's records whose taxon ID is 10239 (virus) and all subtaxa. For instance:
From:
>gi|40555938|ref|NC_005309.1| Canarypox virus, complete genome
To:
>ti|44088|gi|40555938|ref|NC_005309.1| Canarypox virus, complete genome

2.2 ...And now let's try without a MySQL database

```
(within pathoscope directory)
$ python pathoscope.py LIB -genomeFile ../data/nt.fasta -taxonIds 10239
--subTax -outPrefix virus
```

Note that in the command line above (--subTax) the extra hyphen is not a typo.

Again, we are asking PathoLib to look into nt.fasta for all sequences whose taxon ID is 10239 (virus), including all the subtending taxonomies according to NCBI's taxonomy tree (--subTax) and create a file with the prefix 'viral'. On the backend, PathoLib is going to remotely connect to NCBI and download taxonomy information to link GI numbers (present in fasta entries within nt.fasta) to taxon ID numbers (TI) and thus be able to select sequence data according to the user's preferences.

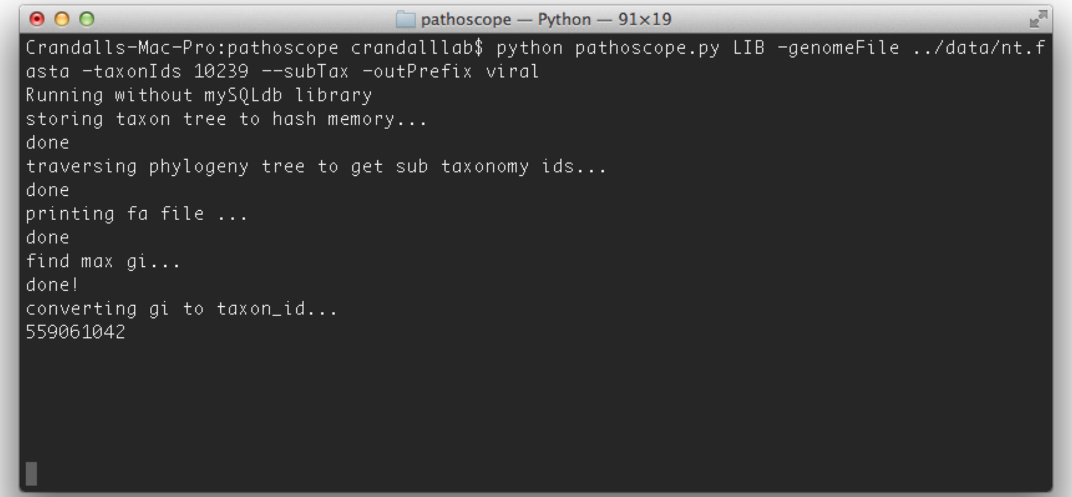

Bear in mind that executing PathoLib can be memory intensive. In order to search the entire GenBank nucleotide database for the taxon IDs you'd like to use for PathoMap, you'd need to allocate something around 6 GB of RAM to PathoLib.

You could decrease memory requirements by using a 2-step approach where 1) you prepend taxIDs to all nt.fasta entries and 2) then you select sequences from the resulting file. Step 1) will be a one time step that you can use as a source to subselect taxa according to your different analyses and needs. Step 1) needs a MySQL database up and running, however, you can download `nt_ti.fa.gz` directly from PathoScope website and start from step 2) without a MySQL database.

Step 1):

```
$ python pathoscope.py LIB -genomeFile ../data/nt.fasta  -outPrefix nt
-dbhost localhost -dbuser pathoscope -dbpasswd johnsonlab
```

Step 2)

```
$ python pathoscope.py LIB -genomeFile nt_ti.fa -taxonIds 2,10239,4751
--subTax -outPrefix microbes
```

This approach will render a file with sequence data for viruses just as the one generated above using a MySQL database. However, downstream your report won't have all the organismal information as it would by using a database. Let's see how to set up a ready-to-use database.

2.3 How to setup a MySQL database

To make things more amenable, we provide a curated version of the nt GenBank database (as of OCT2013) as a MySQL database. Please go to our website and download `pathodb.sql`. Make sure you have MySQL and python package MySQLdb up and running, then simply follow the instructions below:

From the terminal:

```
mysql -u root -p
<Enter root password>
create DATABASE pathodb;
create user pathoscope;
grant all privileges on pathodb.* to pathoscope@"localhost" identified by
'johnsonlab';
flush privileges;
```

And then...

```
mysql -u pathoscope -p pathodb < pathodb.sql
<Enter the following password when asked>
johnsonlab
```

So when you use the PathoLib you should provide your database credentials as follows...

```
$ python pathoscope.py LIB -genomeFile ../data/nt.fasta -taxonIds 10239
--subTax -dbhost localhost -dbuser pathoscope -dbpasswd johnsonlab -out-
Prefix virus_sql
```

3. The PathoMap module

This module will take your reads and map them against a *target library*. Then it will map the mapping reads against a *filter library*. All the reads that map to the *filter library* with a score equal or greater than the mapping score to the *target library* will be discarded. The reads mapping with a better score to the *target library* will be used downstream by the PathoID module.
This computational subtraction mapping approach starts by building genome indices for your target and filter libraries, spliting the libraries as necessary to comply to Bowtie2 architecture (in files of up to 4.3 Gb; Step 1). Then, PathoMap is going to map your reads (fastq) to whatever indices and create a single merged sam file (Step 2). The resulting file will be a filtered sam file, which serves as input for PathoID.

Diagram 2:

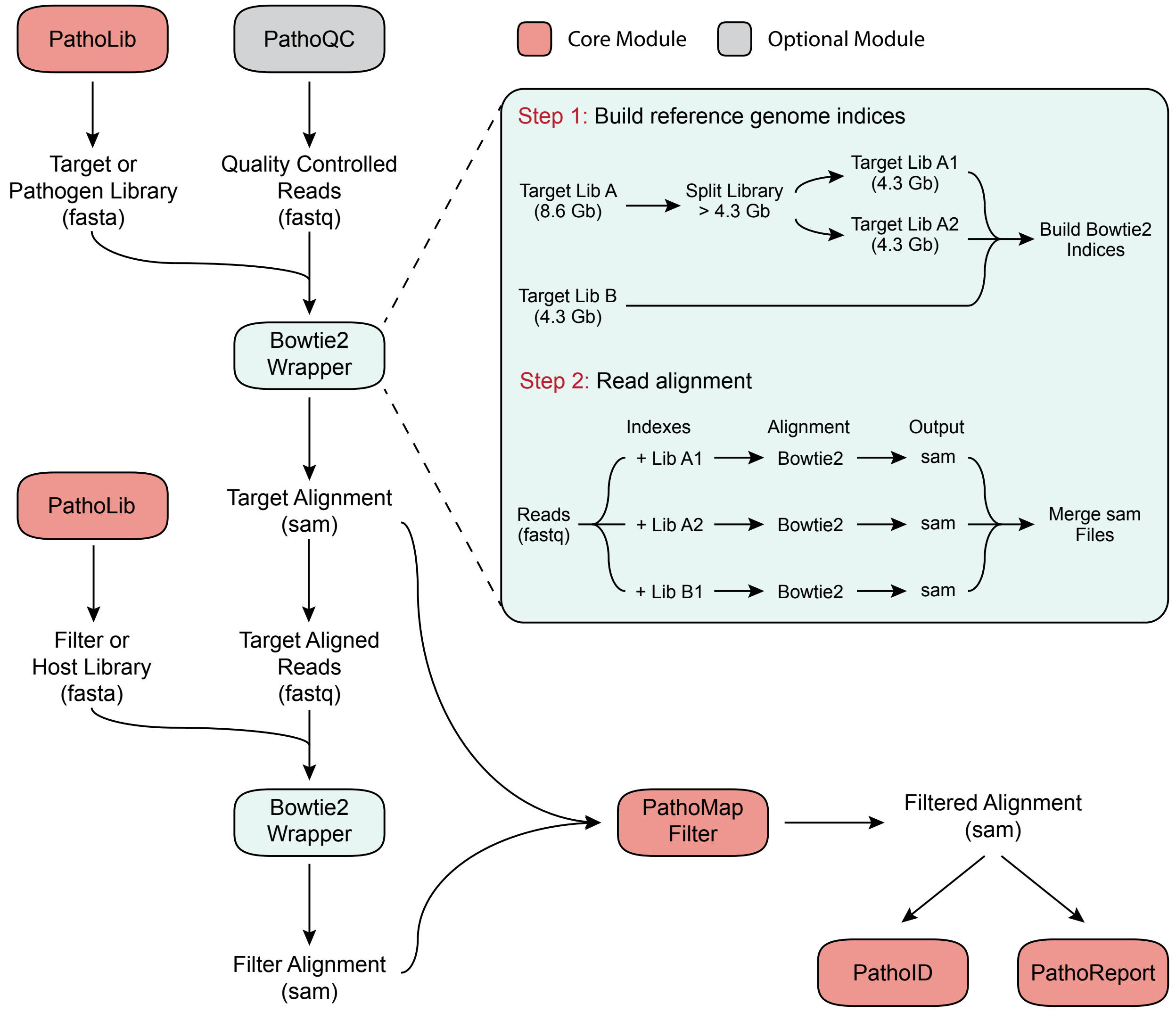

As we stated previously, we will be using reads derived from a Titi Monkey outbreak of unknown viral origin (*Chen et al. 2012*). Here, we are asking what kind of virus is likely responsible for the outbreak by mapping all the reads against our *target library*. This library contains all the sequence data from GenBank under the virus taxonomy ID and it was obtained using PathoScope's library module. Originally, the authors didn't know the etiologic agent of the outbreak. Our case is different because we have included the culprit's genome in the target library. Let's see how it goes.

First, let's map the reads from SRR167721 to our Target library...

```
$ python pathoscope.py MAP -U ../data/SRR167721.fastq -targetRefFiles
viral -filterRefFiles human.fa,phix174.fa  -outDir ../results -outAlign
SRR167721.sam  -expTag tutorial
```

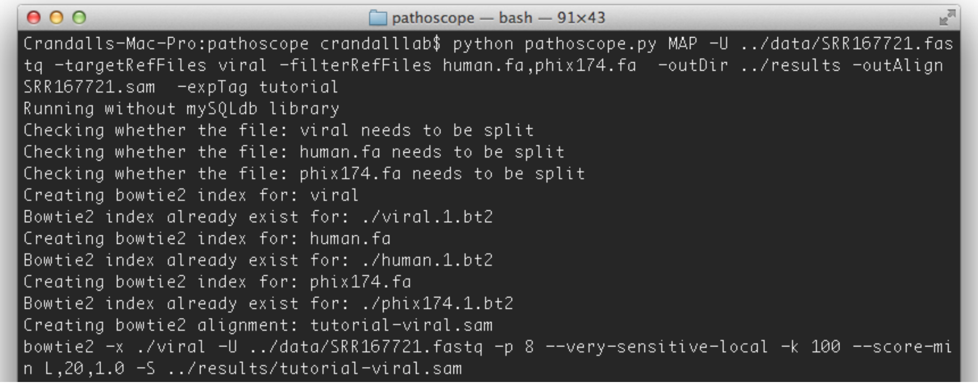

Let's dissect the process. We told PathoMap that our input file is SRR167721.fastq (`-U`), we indicated our Target and Filter files (`-targetRefFiles`, `-filterRefFiles`), and output directory (`-outDir`), filename (`-outAlign`) and experiment tag (`-expTag`). PathoMap is designed so that you could 'enter' it at any point. For example, if you already have indices built, you could simply specify their prefixes (`-targetIndexPrefixes`, `-filterIndexPrefixes`), or if you already have alignment files (sam) for your data against Target and Filter libraries you could start from there as well (`-targetAlignFiles`, `-filterAlignFiles`).

In this example, we use `-U` option to specify our input file. If you have paired-end data, you probably want to use `-1` and `-2` options, as in the example below:

```
$ python pathoscope.py MAP -1 ../data/SRR167721_R1.fastq -2 ../
data/SRR167721_R2.fastq -targetRefFiles viral -filterRefFiles human.
fa,phix174.fa  -outDir ../results -outAlign SRR167721.sam  -expTag tuto-
rial
```

As you can see from the screenshot above, PathoMap is checking whether our *target* and *filter* libraries need be split and whether there are indices built, to then proceed with the mapping. By default, Bowtie2 settings are -p8 --very-sensitive-local -k 100 --score-min L,0,1.2. Basically a score-min of L,0,1.2 corresponds to about 90% identity Score = 0.9 * 2 * L - 0.1 * 6 * L = 1.2 L. In order to give some leeway for the case when there is a gap which has a penalty of 11, and to have some minimum cutoff etc., we selected the above default setting, which should work in most of the cases. There is an option for the user to provide custom bowtie2 parameters, if the user wishes to override the default.

Now, our reads file SRR167721.fastq had an overall aligning rate of 5.56%, 77.46%, and 0.00% against viral, human, and PhiX174 databases, respectively. Let's move on to PathoID!

4. The PathoID module

Diagram 3:

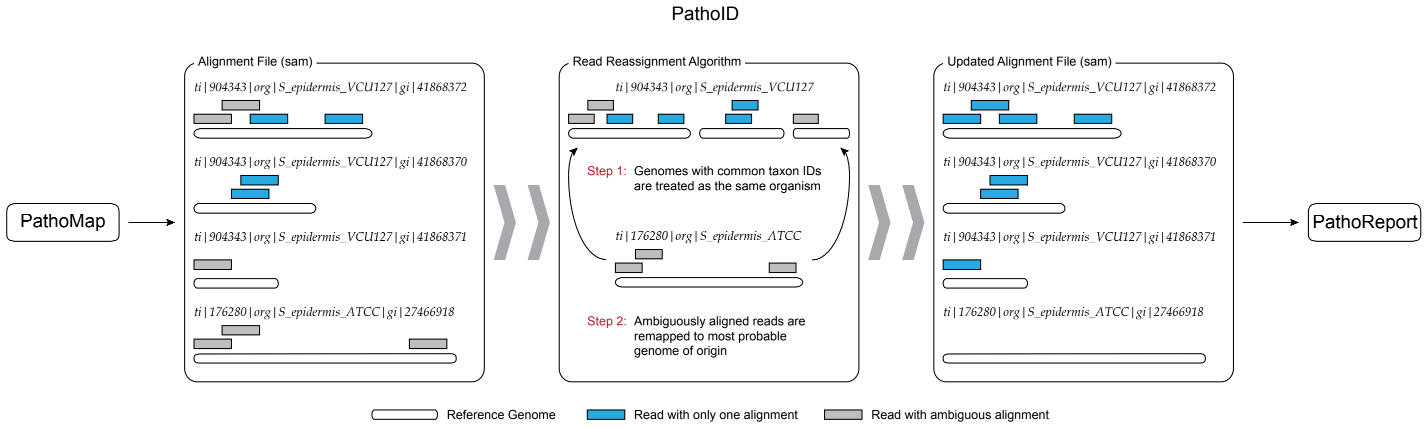

The PathoID module is the core of the PathoScope pipeline. We have seen dramatic increases in specificity and sensitivity due to PathoID Bayesian read reassignment model (*Francis et al. 2013*) over other methods. The key is to identify reads with unique alignments and use them to guide the reassignment of reads with ambiguous alignments (diagram 3).

Let's try PathoID with our file SRR167721.sam generated previously with PathoMap. Try something like the command below (Check additional options with `python pathoscope.py ID -h`)

```
$ python pathoscope.py ID -alignFile ../results/SRR167721.sam -fileType
sam -outDir ../results -expTag tutorial
```

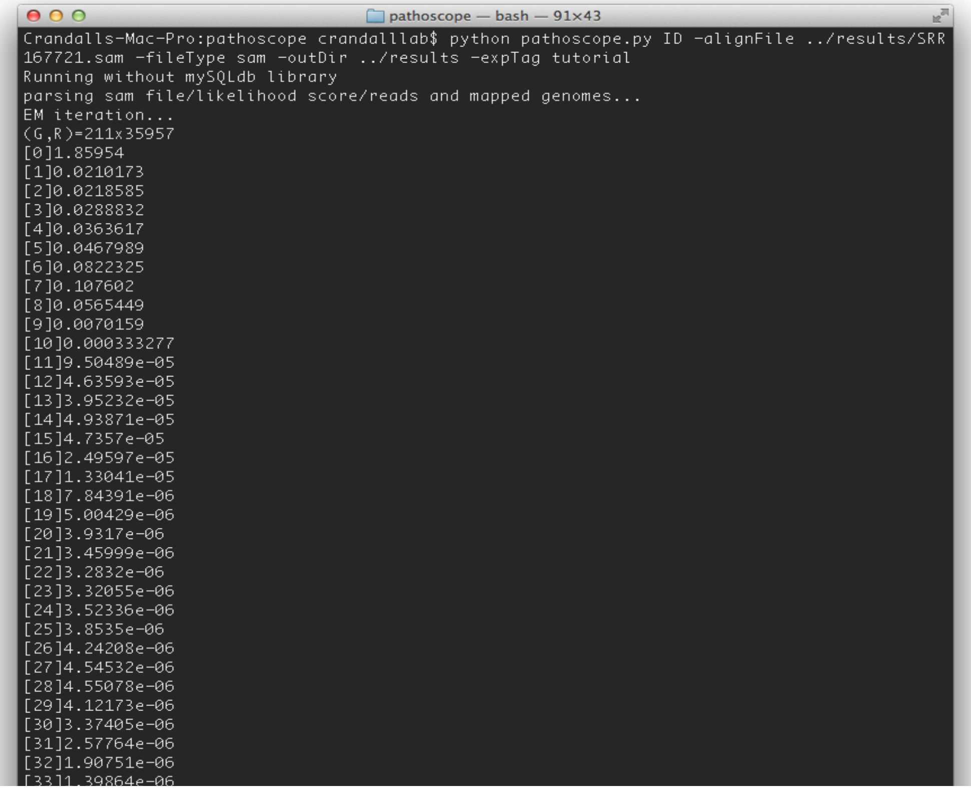

PathoID is parsing SRR167721.sam and using an Expectation-Maximization (EM) algorithm to search for the best parameter estimates (Maximum a posteriori; MAP) for the proportion of mapped reads (pi; theta). PathoID prior distributions and starting values are meant to work for most situations, however, you could use the arguments `-piPrior` and `-thetaPrior` to modify these. Also, to control the parameters of the EM algorithm you could do so by using `-emEpsilon`, `-maxIter`, and `-scoreCutoff` options.

Let's look at our outfile from PathoID. Go ahead and open tutorial-sam-report.tsv in a text editor or spreadsheet software.

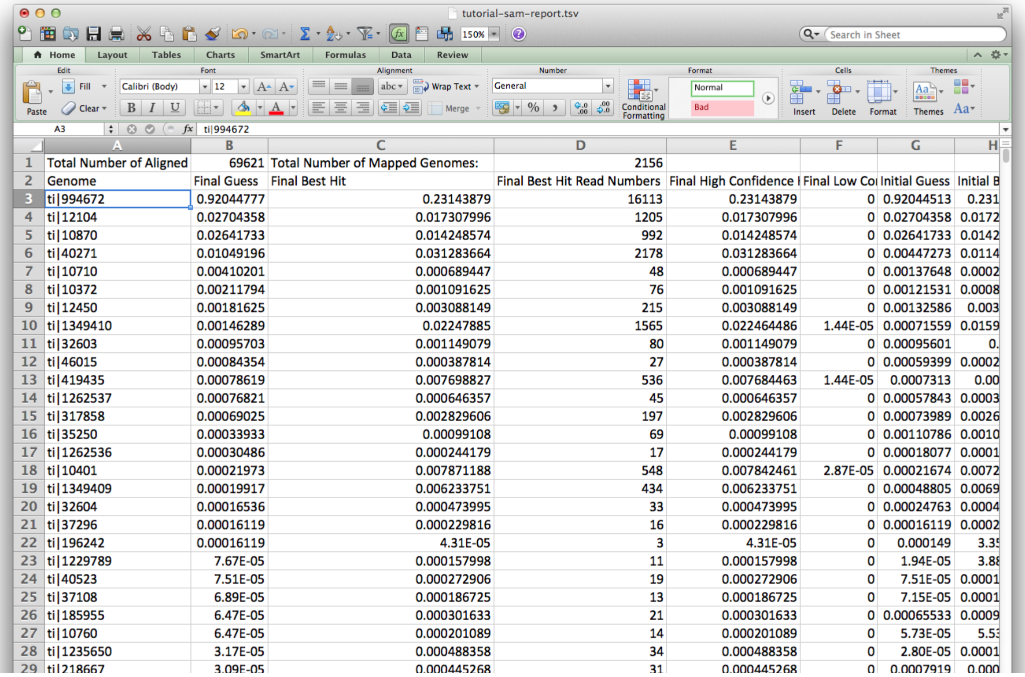

	A	B	C	D	E	F	G	H
1	Total Number of Aligned	69621	Total Number of Mapped Genomes:	2156				
2	Genome	Final Guess	Final Best Hit	Final Best Hit Read Numbers	Final High Confidence I	Final Low Co	Initial Guess	Initial B
3	ti|994672	0.92044777	0.23143879	16113	0.23143879	0	0.92044513	0.231
4	ti|12104	0.02704358	0.017307996	1205	0.017307996	0	0.02704358	0.0172
5	ti|10870	0.02641733	0.014248574	992	0.014248574	0	0.02641733	0.0142
6	ti|40271	0.01049196	0.031283664	2178	0.031283664	0	0.00447273	0.0114
7	ti|10710	0.00410201	0.000689447	48	0.000689447	0	0.00137648	0.0002
8	ti|10372	0.00211794	0.001091625	76	0.001091625	0	0.00121531	0.0008
9	ti|12450	0.00181625	0.003088149	215	0.003088149	0	0.00132586	0.003
10	ti|1349410	0.00146289	0.02247885	1565	0.022464486	1.44E-05	0.00071559	0.0159
11	ti|32603	0.00095703	0.001149079	80	0.001149079	0	0.00095601	0.
12	ti|46015	0.00084354	0.000387814	27	0.000387814	0	0.00059399	0.0002
13	ti|419435	0.00078619	0.007698827	536	0.007684463	1.44E-05	0.0007313	0.00
14	ti|1262537	0.00076821	0.000646357	45	0.000646357	0	0.00057843	0.0003
15	ti|317858	0.00069025	0.002829606	197	0.002829606	0	0.00073989	0.0026
16	ti|35250	0.00033933	0.00099108	69	0.00099108	0	0.00110786	0.0010
17	ti|1262536	0.00030486	0.000244179	17	0.000244179	0	0.00018077	0.0001
18	ti|10401	0.00021973	0.007871188	548	0.007842461	2.87E-05	0.00021674	0.0072
19	ti|1349409	0.00019917	0.006233751	434	0.006233751	0	0.00048805	0.0069
20	ti|32604	0.00016536	0.000473995	33	0.000473995	0	0.00024763	0.0004
21	ti|37296	0.00016119	0.000229816	16	0.000229816	0	0.00016119	0.0002
22	ti|196242	0.00016119	4.31E-05	3	4.31E-05	0	0.000149	3.3[illegible]
23	ti|1229789	7.67E-05	0.000157998	11	0.000157998	0	1.94E-05	3.8[illegible]
24	ti|40523	7.51E-05	0.000272906	19	0.000272906	0	7.51E-05	0.0001
25	ti|37108	6.89E-05	0.000186725	13	0.000186725	0	7.15E-05	0.0001
26	ti|185955	6.47E-05	0.000301633	21	0.000301633	0	0.00065533	0.0009
27	ti|10760	6.47E-05	0.000201089	14	0.000201089	0	5.73E-05	5.5[illegible]
28	ti|1235650	3.17E-05	0.000488358	34	0.000488358	0	2.80E-05	0.0001
29	ti|218667	3.09E-05	0.000445268	31	0.000445268	0	0.0007919	0.000

The first row in this file tells you how many reads were aligned and how many genomes were mapped. In our example above, you can see that 69,621 reads (out of 12.4 Million; 0.56%) mapped 2,156 genomes. Then, from row 2 down you have a ranking of hits ordered ascendently according to the proportion of mapped reads (Final Guess).

'Final guess' represent the proportion of reads that are mapped to the genome in Column 1 (reads aligning to multiple genomes are assigned proportionally) after pathoscope reassignment is performed.

'Final Best Hit' represents the percentage of reads that are mapped to the genome in Column 1 after assigning each read uniquely to the genome with the highest score and after pathoscope reassignment is performed.

'Final Best Hit Read Numbers' represent the number of best hit reads that are mapped to the genome in Column 1 (may include a fraction when a read is aligned to multiple top hit genomes with the same highest score) and after pathoscope reassignment is performed.

In our example above, you can see that all the genomes are coded according to their taxon ID as in GenBank taxonomy database. When you use PathoDB in combination with PathoReport, you get richer and more informative outfiles that include lineage information at different taxonomic levels.

As you can see most of the reads from SRR167721.fastq (92%) mapped Titi Monkey Adenovirus ECC-2011 as reported by *Chen et al. 2011*. The second best hit only gets 2.7% of mapped reads, making it obvious that the dominant viral species is Titi Monkey Adenovirus.

5. PathoReport

This module will make use of the inferences from PathoID combined to organismal information from MySQL database to produce rich XML reports. Just check out PathoReport options by issuing:

```
$ python pathoscope.py REP -h
```

```
Sample_199 — ecastron@login3:~/compute/asthma/pathoscope — ssh — 79×28
[ecastron@login3 pathoscope]$ python2.7 pathoscope.py REP -h
Running without mySQLdb library
usage: pathoscope.py REP [-h] [-samtoolsHome REP_SAMTOOLSHOME]
                         [-dbhost REP_DBHOST] [-dbport REP_DBPORT]
                         [-dbuser REP_DBUSER] [-dbpasswd REP_DBPASSWD]
                         [-db REP_DB] [-outDir REP_OUTDIR] [--contig] -samfile
                         REP_ALI_FILE

optional arguments:
  -h, --help            show this help message and exit
  -samtoolsHome REP_SAMTOOLSHOME
                        Full Path to samtools binary directory (Default: Uses
                        samtools in system path)
  -dbhost REP_DBHOST    specify hostname running mysql if you want to use
                        mysql instead of hash method in mapping gi to taxonomy
                        id
  -dbport REP_DBPORT    provide mysql server port if different from default
                        (3306)
  -dbuser REP_DBUSER    user name to access mysql
  -dbpasswd REP_DBPASSWD
                        provide password associate with user
  -db REP_DB            mysql pathoscope database name (default: pathodb)
  -outDir REP_OUTDIR    Output Directory
  --contig              Generate Contig Information (Needs samtools package
                        installed)
  -samfile REP_ALI_FILE
                        SAM Alignment file path
```

PathoReport applies a cutoff based on when the level1 (>= 0.5) and level2 (0.01 < level2 < 0.5) best hit reads proportion for a genome falls to 0 and when the final proportion of genomes is less than 1%. This is meant to reduce noise in the results that comes from genomes whose read proportions are extremely minor, so the final report is cleaner. Also remember that as in most analyses of sequence data, the idea of Garbage In, Garbage Out (GIGO) holds true for PathoScope. We have developed another optional module, PathoQC, that uses some third-party software to perform automatic quality control on your raw fastq files. Please check it out at http://jlab.bu.edu/software/.

6. Optional Modules

We have implemented three optional modules that complement the core modules described above. These are PathoDB (handles connectivity between MySQL and PathoScope), PathoQC (wraps up third-party read quality control programs [FastQC, cutadapt, and prinseq-lite]), and PathoReport (generates rich xml reports including lineage, gene loci, protein products).

7. Run Times

Run times will obviously vary according to the computer power of the machine one's using. In this tutorial, we have used a MacPro Mid 2010, 2 x 2.4 GHz Quad-Core Intel Xeon, 48 GB 1066 MHz DDR3 ECC, on OS X 10.9 Mavericks. The runtime for PathoLib was < 45 minutes, PathoMap ~30 minutes, and PathoID < 5 minutes. We have randomly subsampled the original fastq file from 12.4 million to 12.4 thousand reads (available here), so that it'll take only 10 minutes the entire process and you still get to practice.

References

Francis, Owen E., Matthew Bendall, Solaiappan Manimaran, Changjin Hong, Nathan L. Clement, Eduardo Castro-Nallar, Quinn Snell et al. "Pathoscope: Species identification and strain attribution with unassembled sequencing data." Genome research 23, no. 10 (2013): 1721-1729.

Chen, Eunice C., Shigeo Yagi, Kristi R. Kelly, Sally P. Mendoza, Nicole Maninger, Ann Rosenthal, Abigail Spinner et al. "Cross-species transmission of a novel adenovirus associated with a fulminant pneumonia outbreak in a new world monkey colony." PLoS pathogens 7, no. 7 (2011): e1002155.

Langmead, Ben, and Steven L. Salzberg. "Fast gapped-read alignment with Bowtie 2." Nature methods 9, no. 4 (2012): 357-359.
